# Identification and characterization of the functional tetrameric UDP-glucose pyrophosphorylase from *Klebsiella pneumoniae*

**DOI:** 10.1128/mbio.02071-24

**Published:** 2024-12-20

**Authors:** Isabel Ramón Roth, Pavel Kats, Timm Fiebig, Françoise Routier, Roman Fedorov, Larissa Dirr, Jana I. Führing

**Affiliations:** 1 Institute of Clinical Biochemistry, Hannover Medical School, Hannover, Germany; 2 Institute for Biophysical Chemistry, Hannover Medical School, Hannover, Germany; 3 Division for Structural Biochemistry, Hannover Medical School, Hannover, Germany; 4 Institute for Biomedicine and Glycomics, Griffith University, Gold Coast, Southport, Australia; Emory University School of Medicine, Atlanta, Georgia, USA

**Keywords:** *Klebsiella pneumoniae*, protein oligomerization, nucleotidyltransferase, UDP-glucose pyrophosphorylase, tetramerization, dimerization, AlphaFold model, structure-function relationship, enzymatic mechanism, antibacterial drug target, *galU*, *galF*

## Abstract

In all kingdoms of life, the enzyme uridine diphosphate-glucose pyrophosphorylase (UGP) occupies a central role in metabolism, as its reaction product uridine diphosphate-glucose (UDP-Glc) is involved in various crucial cellular processes. Pathogens, including fungi, parasites, and bacteria, depend on UGP for the synthesis of virulence factors; in particular, various bacterial species utilize UDP-Glc and its derivatives for the synthesis of lipopolysaccharides, capsular polysaccharides, and biofilm exopolysaccharides. UGPs have, therefore, gained attention as anti-bacterial drug target candidates, prompting us to study their structure-function relationships to provide a basis for the rational development of specific inhibitors. UGP function is tied to its oligomeric state, and the majority of bacterial homologs have been described as tetramers encoded by the *galU* gene. Uniquely, enterobacterial species harbor a second gene, *galF*, encoding a protein with high homology to UGP, whose function is somewhat controversial. Here, we show that the *galF* gene of the opportunistic pathogen *Klebsiella pneumoniae* encodes a dimeric protein that has lost UGP activity, likely due to a combination of active site mutations and an inability to tetramerize, whereas the functional *K. pneumoniae* UGP, encoded by *galU*, is an active tetramer. Our AlphaFold-assisted structure-function relationship studies underline that tetramerization is essential for bacterial UGP function and is facilitated by a common mechanism utilizing conserved key residues. Targeting the respective molecular interfaces, which are absent in human UGP, could provide a means of selectively inhibiting the bacterial virulence factor UGP and potentially rendering pathogenic species avirulent.

**IMPORTANCE:**

The enzyme uridine diphosphate-glucose pyrophosphorylase (UGP) is important for the virulence of bacterial pathogens and, therefore, a potential drug target. In this study, we identify the gene encoding the functional UGP in *Klebsiella pneumoniae*, a bacterium notoriously causing severe antibiotic-resistant infections in humans, and reveal structural and functional features that may aid in the development of new antibiotics.

## INTRODUCTION

The widespread use of antibiotics both in human medicine and animal husbandry has led to the rise of multidrug-resistant bacteria, of which the ESKAPE pathogens (*Enterococcus faecium*, *Staphylococcus aureus*, *Klebsiella pneumoniae*, *Acinetobacter baumannii*, *Pseudomonas aeruginosa*, and *Enterobacter* spp.) are associated with the highest risk of mortality (1, 2). One member of the ESKAPE group is the Gram-negative opportunistic pathogen *K. pneumoniae*, which is able to develop antibiotic resistance by expressing extended-spectrum β-lactamases and carbapenemases (1, 3). *K. pneumoniae* is commonly found in habitats such as soils and surface waters and may asymptomatically colonize the human gastrointestinal tract and nasopharynx (4). However, it can also be found in hospital environments, where its ability to form biofilms enables it to adhere to medical devices, leading to nosocomial infections, including urinary tract infections, pneumonia, and bacteremia (reviewed in reference 5). *K. pneumoniae* is not only detrimental for hospitalized and immunocompromised patients, but the increasing emergence of hypervirulent strains also poses a threat to otherwise healthy individuals (6). At present, there are no approved vaccines available, and existing antibiotic treatments are becoming less effective (7); for example, the Centers for Disease Control and Prevention (CDC) reported that about one-quarter of *K. pneumoniae* infections in the United States in 2021 was resistant to the β-lactam cephalosporin and ca. 14% were multi-drug resistant (8). In the European Union (EU), approximately one-third of infections reported in 2022 was resistant to third-generation cephalosporins, and 20% showed multi-drug resistance (9). These alarming numbers underline the importance of developing new antibacterial strategies. Of note, drugs targeting Gram-negative pathogens such as *K. pneumoniae* face the additional challenge of having to cross two cell membranes with notoriously low permeability, although progress is being made in defining drug design guidelines for overcoming these barriers (reviewed in reference 10) or utilizing specific uptake mechanisms, for example by coupling drugs to siderophores (reviewed in reference 11).

The main virulence factors of *K. pneumoniae* are the capsular polysaccharide (CPS), lipopolysaccharide (LPS), siderophores, and fimbriae (5). Among them, the CPS stands out, as acapsular *K. pneumoniae* strains are dramatically less virulent in mouse models (12–16) and are more likely to be phagocytosed by innate immune cells both in the presence and absence of opsonins (17). The CPS and O-antigen of the LPS are the main factors protecting *K. pneumoniae* from complement attack by binding and sequestering components of the complement system or preventing binding to the bacterial surface altogether (18–20). The presence of CPS is also required for *K. pneumoniae* cytotoxicity, which is caused by isolates expressing different amounts of CPS and/or different serotypes but not by non-encapsulated bacteria (21). Therefore, inhibition of CPS and LPS O-antigen synthesis presents a promising therapeutic approach, whereby the drastic reduction of *K. pneumoniae* virulence ideally leads to a non-pathogenic bacterium (14, 15).

**Fig 1:**
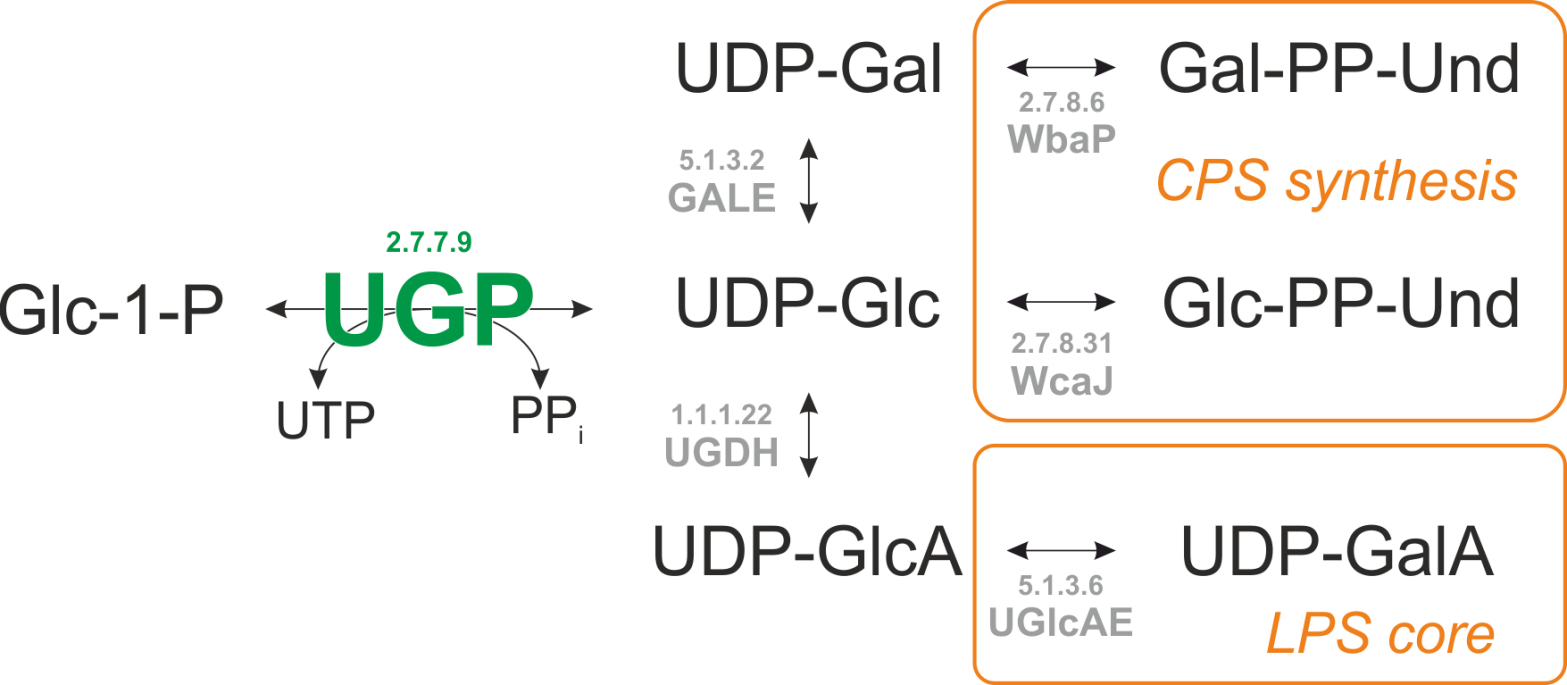
Synthesis and utilization of UDP-Glc in *K. pneumoniae*. UGP converts Glc-1-P and UTP to UDP-Glc and PPi. UDP-Glc can be further converted to UDP-GlcA by UGDH (UDP-glucose 6-dehydrogenase), which can be epimerized to UDP-GalA by UGlcAE (UDP-glucuronate 4-epimerase). UDP-Glc can likewise be epimerized to UDP-Gal by GALE (UDP-galactose-4-epimerase). UDP-Glc and UDP-Gal, respectively, serve as substrates for the initiating CPS GTs WcaJ (undecaprenyl phosphate glucose phosphotransferase) and WbaP (undecaprenyl phosphate galactose phosphotransferase), which transfer the sugar-1-phosphate moiety from the respective UDP-sugar onto undecaprenyl phosphate, yielding Glc-PP-Und (glucosyl-diphospho-undecaprenol) and Gal-PP-Und (galactosyl-diphospho-undecaprenol), respectively. EC (enzyme commission) numbers are denoted above the enzymes.

A common denominator of *K. pneumoniae* CPS and LPS synthesis is the nucleotide sugar uridine diphosphate-glucose (UDP-Glc). *K. pneumoniae* LPS is composed of lipid A, a core oligosaccharide, and a serotype-specific O-antigen. Both types 1 and 2 LPS cores contain a galacturonic acid (GalA) moiety (22–26) which is added from the activated precursor UDP-galacturonic acid (UDP-GalA), a derivative of UDP-Glc (Fig. 1). Furthermore, in all *K. pneumoniae* serotypes, regardless of their specific capsule composition (i.e., K-type), CPS synthesis begins on the cytoplasmic face of the inner membrane by an initiating glycosyltransferase (GT), WcaJ or WbaP. This GT transfers the sugar-1-phosphate moiety from UDP-Glc or its derivative UDP-galactose (UDP-Gal) onto an undecaprenyl phosphate carrier (Fig. 1) (27–31). The enzyme UDP-glucose pyrophosphorylase (UGP, EC 2.7.7.9) catalyzes the reaction of glucose-1-phosphate (Glc-1-P) and uridine triphosphate (UTP) to form UDP-Glc and inorganic pyrophosphate (PPi), the former of which is the precursor of both UDP-Gal and UDP-glucuronic acid (UDP-GlcA; Fig. 1), making UGP a *sine qua non* requirement for both LPS core and CPS synthesis and therefore a candidate drug target. Indeed, *K. pneumoniae* strains lacking a functional UGP show altered CPS and LPS structures, are avirulent to mice, and are highly sensitive to human serum (13, 20). Similar phenotypes have been reported for the ESKAPE pathogen *P. aeruginosa*, where UGP-deficient strains are highly sensitive to human serum and show attenuated virulence in murine infection models (32) as well as reduced cytotoxicity against human lung cells and tissue (33). Overall, impaired UGP function in bacteria has been reported to be associated with various phenotypes such as reduced adherence to cells and tissues (34–39), reduced biofilm formation (35, 39), increased sensitivity to detergents and antimicrobial peptides (35, 37, 40–43) or environmental stress factors (44, 45), impaired motility and intracellular survival (36, 41, 42, 46), and reduced colonization and virulence (34, 36, 39, 43, 47–50).

Functional bacterial UGPs are commonly encoded by the *galU* gene. However, *K. pneumoniae*, like other enterobacteria, additionally harbors a *galF* gene in its CPS gene cluster that encodes a protein with 60% sequence identity to the one encoded by *K. pneumoniae galU*. An involvement of *galF* in CPS synthesis has been postulated for *K. pneumoniae* (51, 52) and *Escherichia coli* (53), although it was shown that the protein encoded by *galF* in *E. coli* (EcGalF) does not exhibit significant UGP activity in itself (54). Instead, it has been proposed to exert a regulatory effect on the *E. coli* UGP enzyme (encoded by *galU*) by interacting with it *in vivo* (55). Therefore, in this study, we first aimed to determine whether *K. pneumoniae galF* and/or *galU* encode an enzymatically active UGP. Second, we aimed to analyze the structure-function relationship of the functional enzyme, which represents a candidate drug target due to its involvement in *K. pneumoniae* CPS and LPS synthesis. Here, we demonstrate that the *galF* gene of *K. pneumoniae* does not encode a functional UGP and investigate the causes for the loss of UGP enzymatic activity. In contrast, *K. pneumoniae galU* encodes a functional UGP that utilizes key molecular interfaces conserved in multiple bacterial species to attain the enzymatically active tetrameric state. These interfaces are essential for forming a functional KpUGP and, consequently, for the synthesis of the *K. pneumoniae* virulence factors CPS and LPS. Therefore, targeting these interfaces to selectively disrupt the active enzymatic species could represent an attractive novel drug development strategy in the fight against multi-resistant bacteria.

## RESULTS

### The *galU* gene encodes the enzymatically active *K. pneumoniae* UGP

Like other enterobacteria, *K. pneumoniae* possesses two candidate genes that could encode a functional UGP: *galU* and *galF*. To investigate which of the two genes encodes the enzyme responsible for producing UDP-Glc in *K. pneumoniae*, we cloned both from genomic DNA into expression vectors, allowing recombinant expression as N-terminally StrepII-tagged (*galU*) or His_6_-tagged (*galF*) proteins of 34.6 and 34.3 kDa size, respectively. The purified *galU* gene product exhibited UGP activity *in vitro* (therefore referred to as KpUGP from here on), whereas the purified recombinant *galF* gene product, KpGalF, did not exhibit significant *in vitro* UGP activity (0.06% of wild-type [wt] KpUGP activity, see Table S1).

### Subunit structure and active site analysis of KpUGP

**Fig 2:**
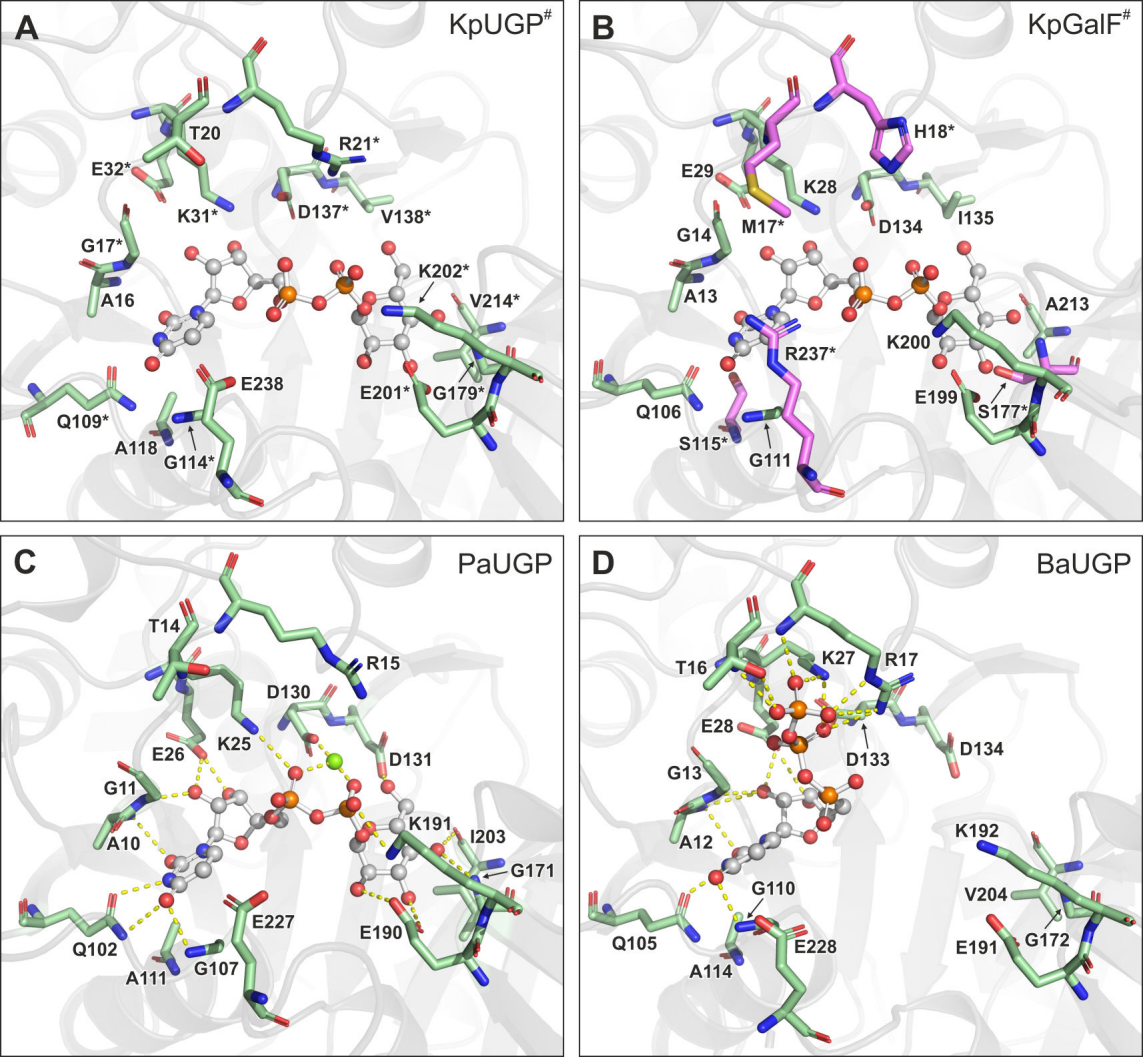
Active site comparison. Active sites of (**A**) KpUGP (AlphaFold model, pTM score: 0.94932), (**B**) KpGalF (AlphaFold model, pTM score: 0.91982), (**C**) PaUGP/UDP-Glc complex (PDB 8F73), and (**D**) *Burkholderia ambifaria* UGP/UTP complex (PDB 5VE7). In panels A and B, UDP-Glc from *Acinetobacter baumannii* UGP (PDB ID 6K8D), which has the highest sequence identity to KpUGP of all bacterial UGPs crystallized in the presence of product, was overlaid onto the active site. Proteins are shown in cartoon representation in gray. Active site residues are shown in stick representation and UDP-Glc/UTP in ball-and-stick representation, with oxygen, nitrogen, phosphorus, and magnesium shown in red, blue, orange, and green, respectively. Protein-substrate/-product interactions of ≤3.3 Å are shown as yellow-dashed lines in panels C and D. Non-conserved residues in KpGalF are highlighted in magenta. * indicates residues mutated in this study, and # indicates AlphaFold models.

To gain insight into the enzymatic mechanism and active site architecture of KpUGP, we generated its AlphaFold model (predicted template modeling [pTM] score: 0.94932; Fig. 2A; Fig. S1B). The predicted tertiary structure of the KpUGP subunit is extremely similar to that of *P. aeruginosa* (Pa)UGP (Fig. S1B and D), which we recently crystallized and characterized in detail (33). Bacterial UGPs share a very similar Rossman-like active site fold based on a central open twisted β-sheet surrounded by α-helices on both sides (Fig. S1B and D). These similarities are consistent with their common ordered sequential Bi Bi catalytic mechanism, in which UTP is the first substrate to enter and UDP-Glc the last product to exit the active site. The only obvious difference between PaUGP and KpUGP is found at the C-terminus, where KpUGP is elongated by 10 residues that form an additional α-helix (Fig. S1A, B, and D).

We recently identified 13 residues involved in substrate binding and/or catalysis in PaUGP (33). Since the majority of these residues are conserved between PaUGP and KpUGP (compare Fig. 2A and C; Fig. S1A), we propose that in KpUGP, the glucose moiety of the product UDP-Glc is coordinated by the sidechain of E201 and the backbones of G179 and V214. The uridyl moiety, in turn, is likely bound by sidechains of E32 and Q109 as well as backbones of A16, G17, and G114. The phosphate groups would be coordinated by sidechains of K31 and K202 as well as a Mg^2+^ ion bound by D137 (Fig. 2A). To assess the importance of these residues for the catalytic activity of KpUGP, we mutated them to alanine and compared the mutants’ activity to that of wt KpUGP, which was defined as 100% (Table 1). Most mutations led to nearly inactive proteins, as expected for essential residues involved in the catalytic center. Determined by classification and regression trees (CART) analysis, the enzymatic activity of the group consisting of Q109A, V138A/D, G179A, K202A, and V214A was significantly less affected with a cut-off at 6% of residual activity (*P* = 0.0012). The most dramatic effect was caused by mutation D137A, underlining this residue’s crucial role in coordinating a Mg^2+^ ion, which is known to be essential for UGP activity (56). In contrast, mutations of G179 (about 80% of wt activity) and V214 (ca. 25%) had a smaller effect on UGP activity, likely because both residues interact with the substrate/product via their backbone, and these interactions can be maintained to some extent in the respective mutants. This finding is supported by the fact that V214 is not strictly conserved across bacterial UGPs (Fig. S2), and we previously showed that the equivalent residue, I203 in PaUGP, is not essential (33).

**TABLE 1:** Enzymatic activity of KpUGP active site mutants^*a*^

Protein	Activity (% of wt)
KpUGP wt	100 ± 3.98
G17A	0.56 ± 0.08
R21A	4.59 ± 0.50
K31A	0.45 ± 0.01
E32A	1.38 ± 0.10
Q109A	8.63 ± 0.88
G114A	1.48 ± 0.09
D137A	0.19 ± 0.01
V138A^*b*^	67.48 ± 11.32
V138D^*b*^	61.14 ± 4.02
G179A	80.04 ± 6.79
E201A	0.67 ± 0.03
K202A	6.00 ± 0.76
V214A^*b*^	25.11 ± 1.29

^*a*^ Activities were determined *in vitro* in the forward reaction (UDP-Glc synthesis) and are given as means ± SEM of at least three independent experiments, each performed in technical triplicate, and expressed as a percentage of wt activity, which was defined as 100%.

^*b*^ Indicates residues not strictly conserved across bacterial UGPs (see Fig. S2).

We also mutated KpUGP V138, which is not conserved across bacterial UGPs (Fig. S2); for example, in PaUGP, an aspartic acid (D131) is found in its place andcontributes to coordinating the glucose moiety (Fig. 2C). Whereas mutation D131A in PaUGP led to an almost inactive protein (33), KpUGP V138A had a residual activity of about 67% of wt, underlining that it does not play an essential role in substrate binding and/or catalysis of KpUGP. We additionally mutated V138 to aspartic acid in an attempt to increase activity due to potentially tighter coordination of the Glc moiety as seen in PaUGP; however, the activity remained at around 61%, suggesting that aspartic acid in this position does not benefit KpUGP activity.

We furthermore mutated R21, whose homolog in other UGPs has been proposed to coordinate UTP in a conformation that exposes the α-phosphate group to the S_N_2 nucleophilic attack by Glc-1-P, as can be observed in the crystal structure of the *Burkholderia ambifaria* UGP/UTP (BaUGP/UTP) complex (PDB 5VE7; Fig. 2D). A KpUGP R21A mutant exhibited only ca. 5% of wt activity, supporting a critical role of this arginine, which is strictly conserved across functional bacterial UGPs (Fig. S2) and even in more distantly related nucleotidyltransferases such as the *P. aeruginosa* Glc-1-P thymidylyltransferase RmlA (57).

### KpGalF active site analysis

We likewise modeled KpGalF using AlphaFold and found its predicted tertiary structure to be highly similar to that of KpUGP and PaUGP (Fig. S1B through D). While the majority of functionally important UGP active site residues are conserved in KpGalF (Fig. S1A), it has previously been proposed for *E. coli* Galf (EcGalF) (54) that the lack of enzymatic activity is caused mainly by the substitution of the catalytic arginine (R21 in KpUGP) by histidine (EcGalF H16/KpGalF H18; Fig. 2A and B; Fig. S1A). The authors further hypothesized that the simultaneous introduction of methionine in the preceding position (EcGalF M15/KpGalF M17), which is occupied by threonine in functional UGPs (T20 in KpUGP; Fig. 2; Fig. S1A), could have an additional deleterious effect. Crystal structures of UTP-bound UGPs from *Acinetobacter baumannii* (6KNJ) and *Burkholderia ambifaria* (5VE7, Fig. 2D) reveal that this threonine is involved in coordinating the UTP γ-phosphate. Indeed, an EcGalF M15T/H16R double mutant exhibited about 10-fold increased UGP activity compared to wt EcGalF (54). We modeled UDP-Glc into the predicted KpGalF structure and noticed that KpGalF S115 is in closer proximity to the uridyl moiety of the superimposed product than the alanine that occupies this position in functional UGPs (A118 in KpUGP; Fig. 2; Fig. S1A). Furthermore, the glycine whose backbone coordinates the Glc moiety (G179 in KpUGP) is replaced by a serine in KpGalF (S177; Fig. 2; Fig. S1A), whose larger sidechain might cause a sterical clash with the sugar ring. Finally, KpGalF R237 (corresponding to KpUGP E238; Fig. 2; Fig. S1A) could affect functionality as it introduces a larger and positively charged sidechain at the opening of the active site. In an attempt to restore the active site architecture (and thus enzymatic activity), we mutated all five aforementioned residues in KpGalF to the respective amino acids found in functional UGPs, i.e., KpGalF M17T/H18R/S115A/S177G/R237E. Although this quintuple revertant mutant was 27-fold more active than wt KpGalF, its activity still only amounted to 1.62% of wt KpUGP activity (Table S1), demonstrating that the loss of UGP enzymatic activity in KpGalF is not exclusively due to the observed amino acid exchanges in the active site. In search of other factors that might contribute to the loss of enzymatic function, we decided to investigate the quaternary structure of both enzymes, since the oligomeric state has been shown to affect the function of bacterial UGPs (33, 54).

### KpUGP quaternary structure analysis

**Fig 3:**
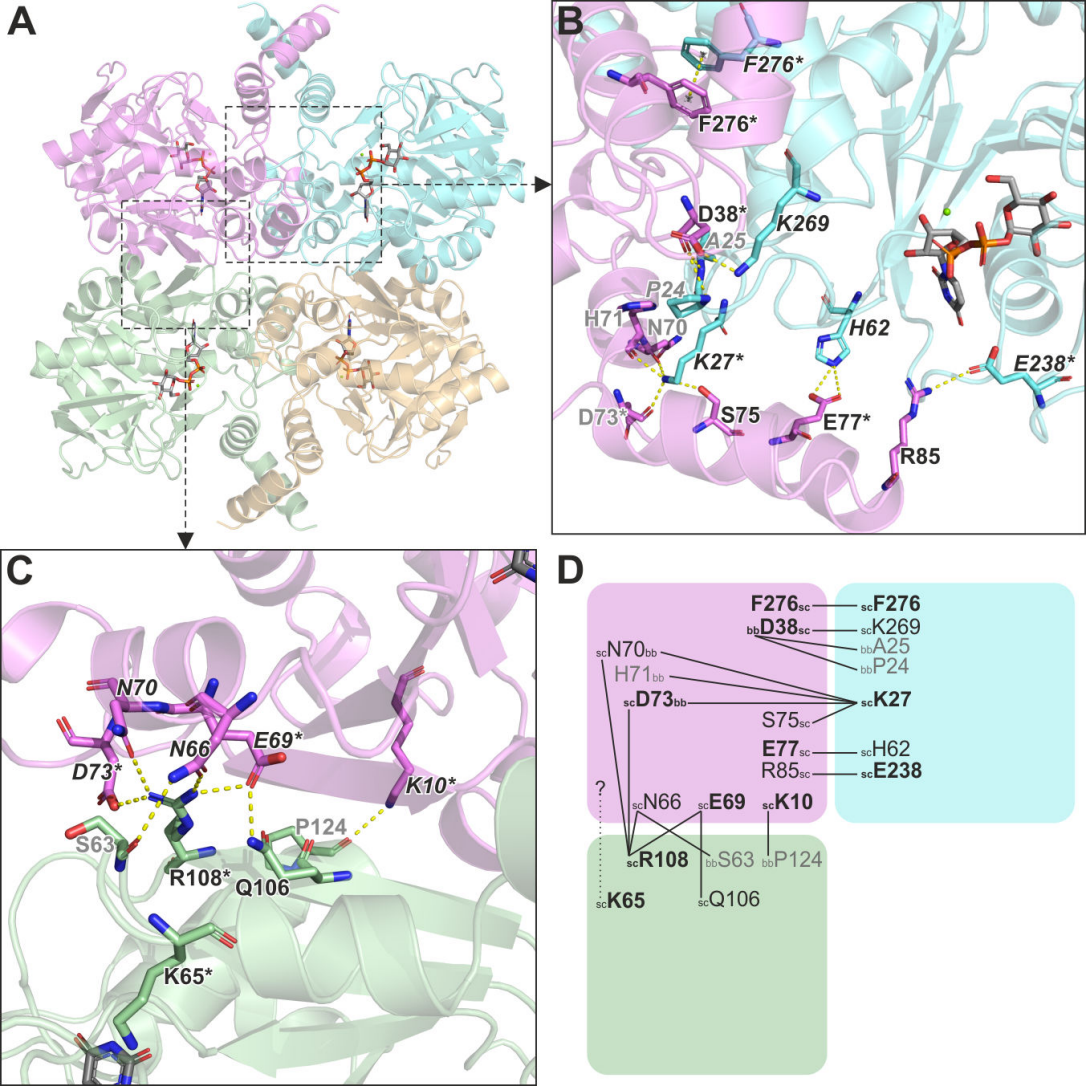
Predicted tetramerization mode of KpUGP. (**A**) AlphaFold model of a tetrameric KpUGP complex shown in cartoon representation with subunits A, B, C, and D shown in magenta, cyan, gold, and green, respectively. UDP-Glc and Mg^2+^ from the PaUGP/UDP-Glc complex (PDB 8F73) were superimposed onto each subunit. (**B**) Tight dimer interface between subunits A and B, and (**C**) loose dimer interface between subunits A and D. Potential interacting residues are shown in stick representation and labeled; asterisks indicate residues mutated in this study. Oxygen, nitrogen, phosphorus, and magnesium are shown in red, blue, orange, and green, respectively. Possible interactions are shown as yellow-dotted lines. (**D**) Schematic depiction summarizing potential interactions, shown exemplarily for chain A, across the KpUGP tight (with chain B) and loose (with chain D) dimer interfaces. Residues predicted to interact exclusively via their backbones are printed in gray. Sc = sidechain and bb = backbone.

**Fig 4:**
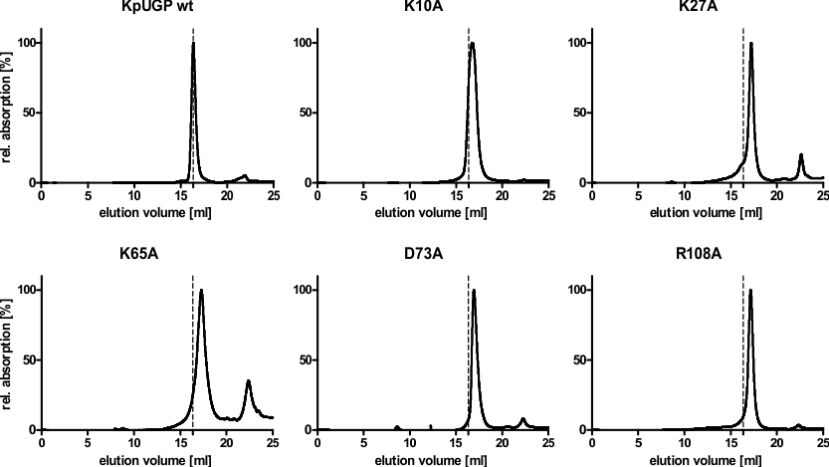
SEC elution profiles of KpUGP wt and mutants with altered oligomeric states. For uniform representation, the maximum peak intensity was set to 100%, resulting in the relative absorption shown on the y-axis. The dashed vertical line corresponds to the elution volume of tetrameric wt KpUGP for reference.

**Fig 5:**
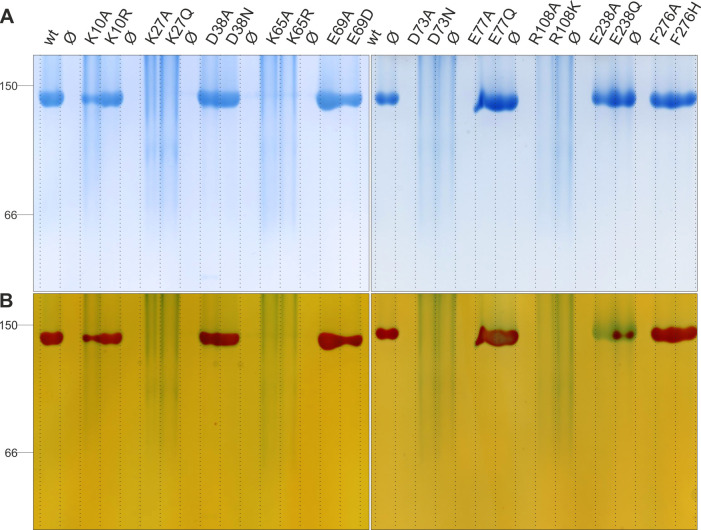
(**A**) BN-PAGE and (**B**) subsequent in-gel activity staining of wt and mutant KpUGP. A total of 4.5 µg of each protein was loaded on a uniform 12% native gel. Electrophoresis was followed by overnight incubation of the gel in a buffer containing the UGP substrates as well as Ca^2+^ ions, causing *in situ* precipitation of the PPi produced by active KpUGP. Alizarin red was used for counterstaining the poorly documentable CaPPi. ∅ Indicates empty lanes.

Nearly all bacterial UGPs have been described to attain homotetrameric assemblies, and we recently showed that the enzymatic function of PaUGP is linked to tetramerization (33). Indeed, KpUGP also eluted as a single peak in size exclusion chromatography (SEC; see Fig. 4 below) corresponding to a calculated oligomeric state of 4.5 (Table 2), indicating a tetrameric assembly. Of note, KpUGP remained stably tetrameric in HEPES-NaOH (pH 8.0) buffer (data not shown), which was shown to promote partial dissociation of *E. coli* UGP (EcUGP) (54). We further confirmed the oligomeric state and enzymatic activity of KpUGP by blue native (BN)-PAGE and subsequent in-gel activity staining (see Fig. 5 below). Based on these experimental findings, we modeled a KpUGP tetramer with AlphaFold Multimer (Fig. 3A). The resulting model (pTM score: 0.94932, interface predicted template modeling [ipTM] score: 0.94349) predicts a dimer of dimers with a “tight” (Fig. 3B) and a “loose” (Fig. 3C) dimer interface, whereby the terminology “tight”/“loose” was adapted from Thoden and Holden (58, 59) and reflects the theoretical buried accessible surface areas at the two dimeric interfaces (“tight”: 2,569 Å^2^ and “loose”: 1,264 Å^2^) calculated for the KpUGP tetramer model. The same mode of assembly was previously described for UGPs from *E. coli* (59), *Corynebacterium glutamicum* (60), *Helicobacter pylori* (61), *Yersinia pestis* (62), and most recently *P. aeruginosa* (33). This prompted us to explore whether, like in PaUGP, enzymatic activity is tied to the tetrameric state.

**TABLE 2:** Enzymatic activity and oligomeric state of KpUGP oligomerization interface mutants^*a*^

Protein	Activity (% of wt)	Elution volume (mL)	Oligomeric state
Wt	100 ± 3.98	16.36	4.5
K10A	49.63 ± 4.07	16.78	3.1
K10R	99.35 ± 3.42	16.35	4.5
K27A	0.87 ± 0.04	17.22	2.1
K27Q	0.10 ± 0.02	17.19	2.2
D38A	63.82 ± 3.20	16.47	4.1
D38N	53.38 ± 4.88	16.43	4.2
K65A	1.58 ± 0.11	17.29	2.0
K65R	1.23 ± 0.04	17.10	2.4
E69A	74.47 ± 3.69	16.39	4.4
E69D	50.48 ± 1.29	16.34	4.6
D73A	18.36 ± 4.25	16.95	2.7
D73N	5.32 ± 0.64	17.10	2.4
E77A	82.92 ± 5.00	16.35	4.5
E77Q	65.81 ± 3.09	16.34	4.6
R108A	0.52 ± 0.02	17.17	2.2
R108K	3.56 ± 0.63	17.08	2.4
E238A	1.91 ± 0.13	16.38	4.4
E238Q	6.33 ± 0.82	16.38	4.4
F276A	112.41 ± 8.15	16.35	4.6
F276H	105.80 ± 6.47	16.35	4.5

^*a*^ Activities were determined in the forward reaction (UDP-Glc synthesis) and are given as means ± SEM of at least three independent experiments, each performed in technical triplicate, and expressed as a percentage of wt activity, which was defined as 100%. SEC was performed using a Superose 6 Increase 10/300 GL column (Cytiva). Oligomeric states were calculated based on elution volumes of proteins of known size.

### Identification of intermolecular contacts in KpUGP

Through analysis of the AlphaFold KpUGP tetramer model, we identified residues that could facilitate contacts between subunits and thereby enable oligomerization. Specifically, we identified 12 residues as potentially involved in the formation of the tight dimer (Fig. 3B and D) and 7 in the formation of the loose dimer (Fig. 3C and D), as well as two residues (N70 and D73) potentially involved in both interfaces (Fig. 3B through D). Of the 21 residues thus identified, 14 are strictly conserved or functionally similar in PaUGP (Fig. S1A), 8 of which were shown to participate in its oligomerization (33) (Fig. S5B and C). In order to study the connection between KpUGP tetramerization and activity, we selected 10 residues that presumably interact via their sidechains (Fig. 3B through D; Fig. S1A) and mutated each of them both to alanine (non-conservatively) and to an amino acid with similar properties (conservatively). Of note, KpUGP K65 was included in the mutagenesis scheme based on the role of its homolog K59 in PaUGP (Fig. S5C) (33), despite not appearing to interact with other subunits in KpUGP (Fig. 3C). All mutant proteins could be obtained with a comparable purity to wt KpUGP. To assess the mutations’ impact on oligomerization and enzymatic activity, we performed SEC (Fig. 4; Table 2; Fig. S6), determined the enzymatic activity *in vitro* relative to wt KpUGP (Table 2), and performed BN-PAGE followed by in-gel activity staining (Fig. 5) to visualize the active enzyme species.

### Tight dimer interface

At the tight dimer interface (Fig. 3B and D), the sidechain of K27 is predicted to be within hydrogen-bonding distance of the backbones of N70, H71, and D73, as well as the sidechain of S75. Therefore, we expected the mutation of K27 to result in the disruption of the tight dimer interface, as previously observed for the homologous residue in PaUGP, K21, which engages in interactions with the PaUGP homologs of H71 and D73 (Fig. S5B) (33). Indeed, KpUGP K27 mutants eluted exclusively (K27A) or predominantly (K27Q) as (presumably loose) dimers in SEC (Fig. 4; Table 2; Fig. S6) and exhibited under 1% of wt KpUGP activity in solution (Table 2). Mutation of K27 likewise led to the complete loss of defined tetramer bands as well as loss of in-gel activity in BN-PAGE (Fig. 5).

D38 presumably interacts with the sidechain of K269 via its sidechain and with the backbones of P24 and A25 via its backbone; the latter two interactions are likewise established in PaUGP by the D38 homolog N32 (Fig. S5B). D38 mutants were tetrameric in SEC and showed between 53% and 64% of wt activity in solution (Table 2; Fig. S6). Similarly, in BN-PAGE, both proteins were stable tetramers and displayed strong activity staining (Fig. 5), indicating that at least D38’s sidechain is not relevant for tetramer stability and has a minor impact on KpUGP activity.

Three further potential interactions, which were not observed in PaUGP, were found between the sidechains of E77 and H62, as well as those of E238 and R85, respectively; finally, F276 could potentially interact with its counterpart from the neighboring subunit via π-stacking. Mutation of E77 and F276 resulted in mostly (E77) or fully (F276) active tetrameric species in solution (Table 2; Fig. S6) and likewise had no impact on tetramer stability and activity in BN-PAGE (Fig. 5), indicating that these residues play a minor (E77) or no (F276) role in oligomerization and activity of KpUGP. In contrast, mutation of E238 resulted in tetramers with very little residual activity (ca. 2 and 6% for E238A and E238Q, respectively; Table 2; Fig. S6). Similarly, both mutants were stable tetramers in BN-PAGE but showed little (E238Q) to no (E238A) in-gel activity (Fig. 5). Given that this residue is located at the edge of the active site cleft, its mutation could—for example—affect substrate access.

### Loose dimer interface

At the loose dimer interface (Fig. 3C and D), we mutated K10, whose sidechain could interact with the backbone of P124. K10A exhibited about 50% of wt activity and displayed a calculated oligomeric state of 3.1 in solution (Table 2), which could correspond to a mixture of tetramer and dimer, supported by the notion of a broadened peak in SEC (Fig. 4) and a smear in BN-PAGE (Fig. 5). This suggests that K10A partially dissociates into (presumably tight) dimers, which is accompanied by a partial loss of KpUGP activity. In contrast, conservative mutation (K10R) results in a fully active tetrameric enzyme both in solution (Table 2; Fig. S6) and in BN-PAGE (Fig. 5), indicating that replacement by another basic amino acid preserves K10’s functional interactions.

Even though, based on the AlphaFold model, it did not appear to interact with a neighboring subunit, we also mutated K65 (Fig. 3C) because its homolog PaUGP K59 was seemingly involved in the network of interactions at the loose dimer face (Fig. S5C). Interestingly, while mutation of the corresponding PaUGP residue caused loss of enzymatic activity without affecting tetramerization (33), KpUGP K65 mutants were fully dissociated and inactive both in solution (Fig. 4; Table 2; Fig. S6) and BN-PAGE (Fig. 5), indicating that the residue engages in interactions that are crucial both for KpUGP tetramer stability and enzymatic function.

The sidechain of E69 likely interacts with the sidechain of R108 (as observed for the homologous PaUGP residues E63 and R101, see Fig. S5C) as well as Q106. Mutation E69A resulted in a tetrameric species that still conserved ca. 75% of wt activity, whereas the conservative mutation E69D was likewise tetrameric but only ca. 50% active (Table 2; Fig. S6). In BN-PAGE, both mutants were tetrameric and active, although E69D appeared to be slightly less stable and less active than E69A (Fig. 5), which is in line with results from the *in vitro* activity assay.

D73 likely interacts (via its sidechain) with the sidechain of R108 across the loose dimer interface, as well as (via its backbone) with the sidechain of K27 at the tight dimer interface, as mentioned above. The same interactions are found in the PaUGP tetramer between the corresponding residues D67, R101, and K21 (Fig. S5B and C). In BN-PAGE, KpUGP D73 (A/N) mutants were dissociated and did not show any activity staining (Fig. 5). In solution, both mutants eluted as (presumably tight) dimers and exhibited an average activity of ca. 18% and 5% for D73A and D73N, respectively (Fig. 4; Table 2; Fig. S6). Upon exemplary closer inspection of D73A, we found that the mutant enzyme rapidly and progressively loses activity during storage on ice (Fig. S7A). The observed, statistically significant loss of activity correlates with progressive dissociation of the mutant protein in solution, as reflected by a shift of the SEC elution profile towards a smaller oligomeric species over time (Fig. S7B).

The sidechain of R108, in addition to the above-mentioned (conserved) interactions with E69 and D73, could engage in additional interactions with the sidechains of N66 and/or N70. R108 mutants were dissociated and inactive in BN-PAGE (Fig. 5) and eluted as (presumably tight) dimers in SEC with *in vitro* activities of 0.5% (R108A) to 3.6% (R108R) of wt KpUGP (Fig. 4; Table 2; Fig. S6).

Determined by CART analysis, the group consisting of K27A/Q, K65A/R, D73A/N, R108A/K, and E238A/Q was significantly more affected by loss of enzymatic activity with a cut-off at 18% of residual activity (*P* < 0.0001). With the exception of E238A/Q, these mutations simultaneously disrupted the native KpUGP tetramer. In summary, our results indicate that tetramerization of KpUGP is facilitated by the key residues K27, K65, D73, and R108, whose mutation caused dissociation into dimers, accompanied by loss of UGP enzymatic activity. These findings suggest that tetramerization is essential for the enzymatic function of KpUGP, as was recently shown for PaUGP (33). Of note, the identified key residues are equivalent to PaUGP K21, K59, D67, and R101 (Fig. S5B and C), which we have previously shown to affect tetramer stability and/or PaUGP activity (33), and are strictly conserved across Gram-negative bacteria (Fig. S2), suggesting that both the mechanism and purpose of oligomerization in bacterial UGPs are conserved.

### KpGalF quaternary structure analysis

Having found that tetramerization of KpUGP is essential for activity, we likewise analyzed the oligomeric state of KpGalF. Interestingly, while EcGalF was described to be monomeric (54), which we confirmed experimentally (data not shown), KpGalF eluted as a dimer in SEC (calculated oligomeric state 1.9, see Table S1) and as a smear, likely corresponding to a dimer-monomer mixture that exhibited no UGP activity staining, in BN-PAGE (Fig. S8). These observations suggest that KpGalF is capable of forming dimers, but not tetramers, and the negligible UGP activity in KpGalF (even after the restoration of the active site) could be linked to the altered quaternary structure, thus further underlining the importance of tetramerization for the activity of bacterial UGPs.

**Fig 6:**
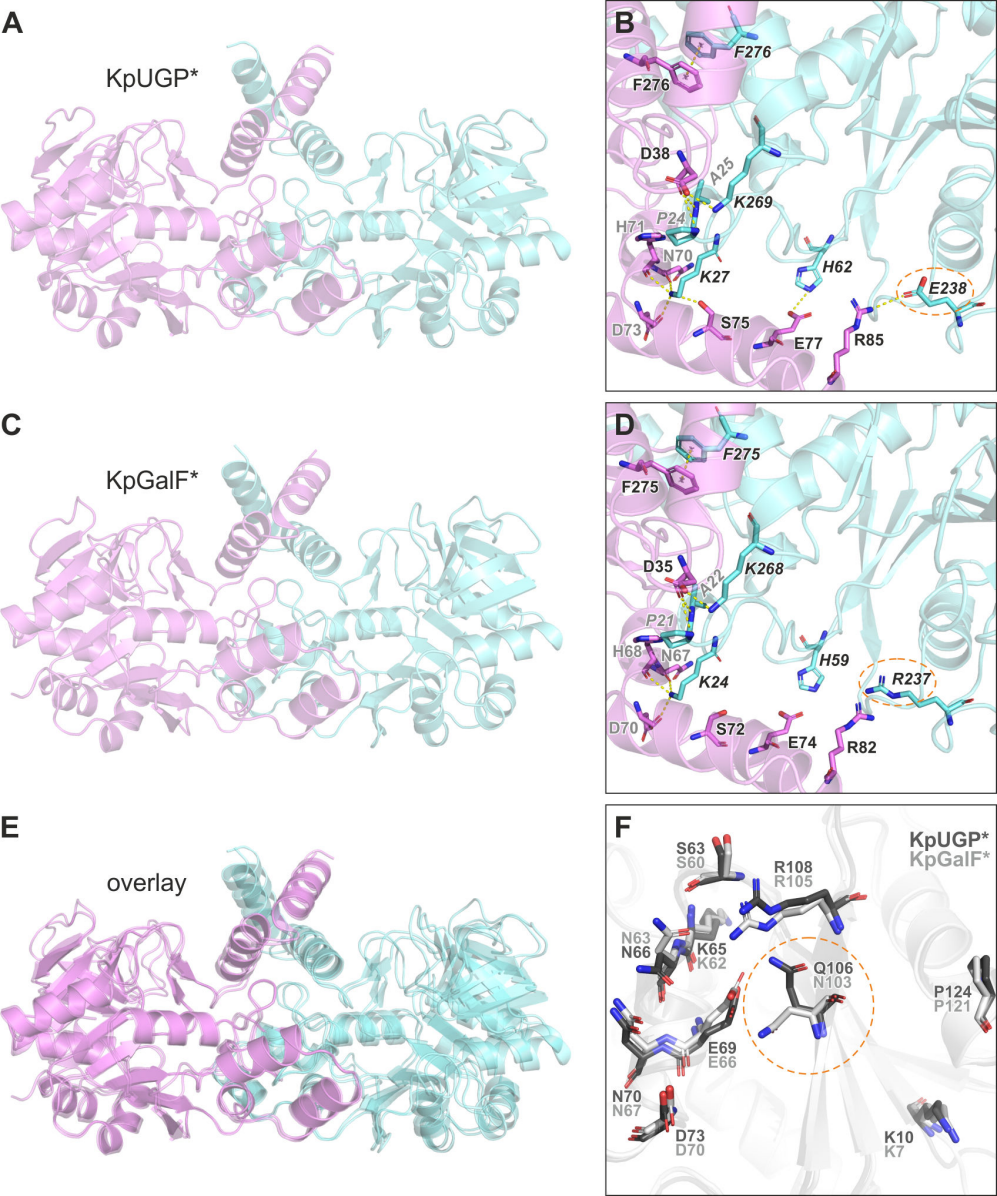
Comparison of predicted KpUGP and KpGalF oligomerization. (**A**) Tight dimer portion of the KpUGP AlphaFold model. (**B**) Close-up view of potential contacts at the KpUGP tight dimer interface. (**C**) Dimeric AlphaFold model of KpGalF. (**D**) Close-up view of potential contacts at the KpGalF tight dimer interface. (**E**) Overlay of KpUGP (tight dimer portion) and KpGalF tight dimer, generated by secondary-structure matching superimposition of chains A (magenta). (**F**) Superimposed chains A of KpUGP and KpGalF illustrating residues potentially involved in KpUGP loose dimer formation and their homologs in KpGalF. Oxygen and nitrogen are shown in red and blue, respectively. Interactions are shown as yellow-dotted lines. Amino acids differing between KpUGP and KpGalF are highlighted with broken orange ellipses.

Based on the protein’s observed dimeric state in solution, we generated a KpGalF dimer model with AlphaFold Multimer (pTM score: 0.91982 and ipTM score: 0.90083), which resembled the tight dimer portion of KpUGP (Fig. 6A, C and E). Upon closer inspection, the majority of predicted interactions at the KpUGP tight dimer interface are conserved in the modeled KpGalF tight dimer, with one major exception: the strong electrostatic interaction between KpUGP R85 and E238 cannot be established in KpGalF since the latter residue is replaced by arginine (R237) in GalF, whose proximity to R82 from the neighboring KpGalF subunit could potentially cause repulsion (Fig. 6B and D). At the loose dimer interface, 9 of 10 potentially interacting KpUGP residues are conserved in KpGalF, the only exception being KpUGP Q106/KpGalF N103 (Fig. 6F).

Out of the two non-conserved amino acids at the tight and loose dimer interfaces (Fig. 6B, D, and F), we speculated that the replacement of KpUGP E238 by arginine in KpGalF would likely be far more disruptive than the replacement of KpUGP Q106 by the smaller asparagine in KpGalF since the latter two both have the same functional terminal primary amide group. Indeed, our KpUGP mutagenesis study suggested that Q106 is not crucial for tetramerization and activity since mutants of its proposed interaction partner E69 were stable tetramers that retained up to 75% of activity. To study the effect of the E238/R237 exchange, we produced a KpUGP E238R mutant. Like the previously created KpUGP E238A and E238Q mutants (Table 2; Fig. S6), E238R was inactive both *in vitro* and in native PAGE (Fig. S9A and B). In BN-PAGE, E238R showed a faint, broad smear likely indicating dissociation, as well as a diffuse signal above the position of tetramer bands, perhaps indicating aggregation under these particular experimental conditions. Of note, whereas E238A and E238Q were stable tetramers in solution, the peak of the E238R mutant was shifted in SEC (Fig. S9C) with a calculated oligomeric state of 3.1, possibly representing a tetramer/dimer mixture. These findings suggest that the replacement of KpUGP E238 by arginine, mimicking the respective constellation in KpGalF, destabilizes the tetramer and abolishes enzymatic activity.

Asking whether, in turn, the reverse mutation of this position in KpGalF would promote tetramerization, we analyzed the oligomeric state of the KpGalF quintuple mutant, which encompassed the reverse mutation R237E. However, the mutant migrated like KpGalF wt in SEC (calculated oligomeric states: 1.7 and 1.9, respectively, see Table S1), suggesting that additional factors contribute to KpGalF’s inability to form tetramers.

Ebrecht and coworkers hypothesized that the monomeric state of EcGalF was caused by differences in the protein’s C-terminus when compared to EcUGP; specifically, the two C-terminal α-helices that are involved in the formation of the tight dimer (54). To investigate the role of these helices in the oligomerization of KpGalF, we removed the latter of the two (∆H2) or both terminal helices (∆H1,H2) or replaced both helices with the respective sequence of tetrameric KpUGP (mutations indicated in Fig. S1). Upon removal of one or both helices, the proteins eluted as monomers (calculated oligomeric states: 1.1 for ∆H2 and 1.2 for ∆H1,H2; see Fig. S10) in SEC, demonstrating that the helices support dimerization and that KpGalF indeed forms tight rather than loose dimers, as predicted by AlphaFold. Exchange of both helices by the respective KpUGP sequence (KpGalF∆H1,H2+KpUGP H1,H2) did not promote the formation of tetramers (Fig. S10), underlining that the differences in this particular section of the structure are not solely responsible for KpGalF’s inability to form tetramers.

Taken together, the results of our mutagenesis studies on both KpUGP and KpGalF underline the functional importance of tetramerization for enzymatic activity of bacterial UGPs. Importantly, we provide additional evidence that the mode of assembly of functional tetrameric UGPs, including key residues mediating the interactions between subunits, is conserved in bacteria. Therefore, the molecular interfaces involved in the tetramerization of bacterial UGPs may be targeted for specific inhibition of UGP in pathogenic species like *P. aeruginosa*, *K. pneumoniae*, and others that require this enzyme for virulence.

## DISCUSSION

The opportunistic pathogen *K. pneumoniae* stands among the most prominent multidrug-resistant bacteria, as it is able to express an extended spectrum of β-lactamases and carbapenemases, making infections increasingly difficult to treat. New strategies are therefore needed to combat this pathogen, ideally by targeting the synthesis of its virulence factors such as CPS or LPS. The nucleotide sugar UDP-Glc and its derivatives UDP-Gal and UDP-GalA are essential substrates in the synthesis of both polysaccharides. Therefore, the UDP-Glc-producing enzyme UGP represents an attractive novel drug target candidate.

*K. pneumoniae* harbors two genes, *galF* and *galU*, that share 60% sequence identity and have both been suggested to possess UGP activity and to be involved in the synthesis of *K. pneumoniae* CPS (20, 52). Here, we determined the ability of both gene products to produce UDP-Glc *in vitro* and found that the *galU* gene product (termed KpUGP) was enzymatically active, whereas the *galF* gene product (KpGalF) had only 0.06% of KpUGP activity, similar to the constellation in *E. coli* where the *galF* gene product had only 0.004% of the *galU* gene product’s activity (54). Ebrecht and colleagues hypothesized that the loss of enzymatic function in EcGalF could be due to mutation of active site residues and/or differences in the quaternary structure between the two proteins (54). Building on this hypothesis, we investigated the contributions of both these factors to the loss of function in KpGalF. As previously described for *E. coli*, a catalytically crucial arginine (R21 in KpUGP) is replaced by methionine in GalF, and the preceding threonine is replaced by histidine in GalF (Fig. S1 and S3) (54). However, upon comparing KpUGP and KpGalF AlphaFold models, we identified three additional substitutions in the active site that likely affect enzymatic function: KpGalF S115 and S177 (replacing A and G, respectively, in UGPs), which could cause sterical hindrance in the uridyl and glucose binding pockets, respectively, and R237 (replacing E in UGPs), which might adopt a conformation that interferes with substrate entry and/or product release. Of note, these five active site residues differing from functional UGPs are conserved across GalF proteins from various enterobacteria, e.g., *K. pneumoniae* and *variicola*, *E. coli*, *Enterobacter cloacae*, *Salmonella enterica*, and *Shigella flexneri* (Fig. S3). A quintuple revertant mutant of KpGalF, created in an attempt to restore the active site architecture and thereby UGP activity, was 27× more active than wt KpGalF, representing a modest improvement compared to the reported double revertant mutant of EcGalF (M15T/H16R), whose activity was 10× higher than wt EcGalF (54). Nevertheless, the activity of the quintuple mutant amounted to only 1.62% of wt KpUGP, suggesting that the drastically diminished activity of KpGalF is caused by additional factors such as the quaternary structure. We show here that KpUGP—like the majority of characterized bacterial UGPs—is a tetramer, whereas KpGalF (as well as its quintuple mutant) is a dimer corresponding to the tight dimer portion of tetrameric UGPs. Importantly, any mutations that disrupted the KpUGP native tetrameric structure—yielding dimeric proteins—were accompanied by a dramatic reduction in activity (between 0% and ca. 3% of wt KpUGP), comparable to the likewise dimeric KpGalF quintuple mutant (ca. 1.6% of wt KpUGP activity). These findings strongly suggest that the drastically reduced activity of KpGalF, which persisted even after the attempted restoration of the active site, is caused by its inability to form tetramers. Ebrecht and coworkers previously showed by phylogenetic analysis that the GalF protein evolved from a *galU*-encoded UGP in enteric bacteria and subsequently acquired mutations that abolished its enzymatic function (54). It is likely that the ability to form tetramers was likewise lost—to different extents, e.g., dimeric KpGalF and monomeric EcGalF—during this evolution.

To understand why KpGalF is incapable of forming tetramers, we compared potential intermolecular interactions between the KpUGP (dimer of dimers) and KpGalF (“tight” dimer) AlphaFold models. Surprisingly, we identified only two differences among the presumably involved residues: KpGalF R237 (E238 in KpUGP) at the tight dimer interface and N103 (Q106 in KpUGP) at the loose dimer interface. We deemed the former far more likely to interfere with oligomerization since its replacement by arginine could abolish a strong electrostatic interaction (R-E) and could introduce repulsion between R82 and R237 (Fig. 6D). Indeed, introducing the corresponding mutation into KpUGP (E238R) caused partial dissociation of the protein and loss of activity. However, reverse mutagenesis of KpGalF R237 to E, which was contained in the quintuple revertant mutant, did not alter the dimeric state of the protein. We furthermore observed a slight tilt in the orientation of chain B relative to chain A in the modeled KpGalF dimer, compared to the KpUGP tight dimer (Fig. 6E), which might be caused at least in part by repulsion between R82 and R237. This tilt might, in turn, hinder the association of two KpGalF tight dimers into a stable tetramer via loose dimer interactions.

Given its largely abolished enzymatic activity, the biological function of KpGalF *in vivo* and its possible involvement in CPS synthesis remain unknown. In line with observations that enzymes may evolve to lose catalytic activity but acquire regulatory functions (63), it was shown that in *E. coli*, GalF (when coexpressed with UGP/*galU*) can modify certain biochemical and physical properties of UGP including thermal resistance, which could be important for bacterial adaptation to conditions of stress (55). The authors showed that in *E. coli*, GalF interacts physically with UGP, suggesting that it acts as a regulatory component or non-catalytic subunit of UGP, although the precise mode of interaction is unknown. It is possible that similar mechanisms exist in *K. pneumoniae*; although, based on our findings, it appears unlikely that KpGalF and KpUGP would be capable of forming stable hetero-oligomers.

The finding that KpUGP (encoded by *galU*) is responsible for providing UDP-Glc in *K. pneumoniae* underlines its status as a drug target candidate. The same holds true for *P. aeruginosa* UGP, whose structure-function relationships we have previously studied in detail (33). Both KpUGP and PaUGP are active tetrameric enzymes, and all mutations that fully disrupted the native tetrameric assembly were accompanied by a dramatic reduction in activity (between 0% and around 5% of the respective wt enzyme), demonstrating that tetramerization is essential for the functionality of both UGPs. Thus, we propose that in both cases, the oligomerization interfaces could be targeted to disrupt the active tetrameric species. Importantly, these interfaces are absent from the human homolog (*Homo sapiens* [Hs]UGP) which forms functional octamers via a C-terminal β-helix domain that exists exclusively in eukaryotic UGPs (Fig. S11) (64, 65). Since it would be particularly attractive to develop small molecules capable of broadly inhibiting bacterial UGPs by targeting conserved sites (which are absent in the host enzyme), we comparatively analyzed the quaternary structures and functional intermolecular interactions in KpUGP and PaUGP.

The predicted and experimental structures suggest that the interactions in KpUGP might be even more extensive than in PaUGP, perhaps additionally aided by an extra α-helix at the C-terminus, which participates in tight dimer formation (Fig. S5A). In line with these observations, the theoretical buried accessible surface areas at the tight and loose dimer interfaces of KpUGP (2,569 and 1,264 Å^2^) were slightly higher than those calculated for PaUGP (2,005 and 1,054 Å^2^, respectively) (33). Furthermore, our insights from PaUGP suggested that KpUGP K65 might participate in a network of electrostatic interactions at the loose dimer interface, and indeed, K65 mutants were dissociated into dimers and nearly inactive. This was in contrast to PaUGP, where mutants of the homologous residue K59 remained tetrameric (33). Interestingly, the KpUGP AlphaFold model did not predict intermolecular contacts for K65 but instead suggested that it could form intramolecular ionic interactions with the active site residue E32, whose mutant showed an almost identical phenotype of inactive dimers (Fig. S4; Table S2). Therefore, it appears that K65 is primarily involved in stabilizing the active site, and the effect on KpUGP tetramer stability may be secondary.

**Fig 7:**
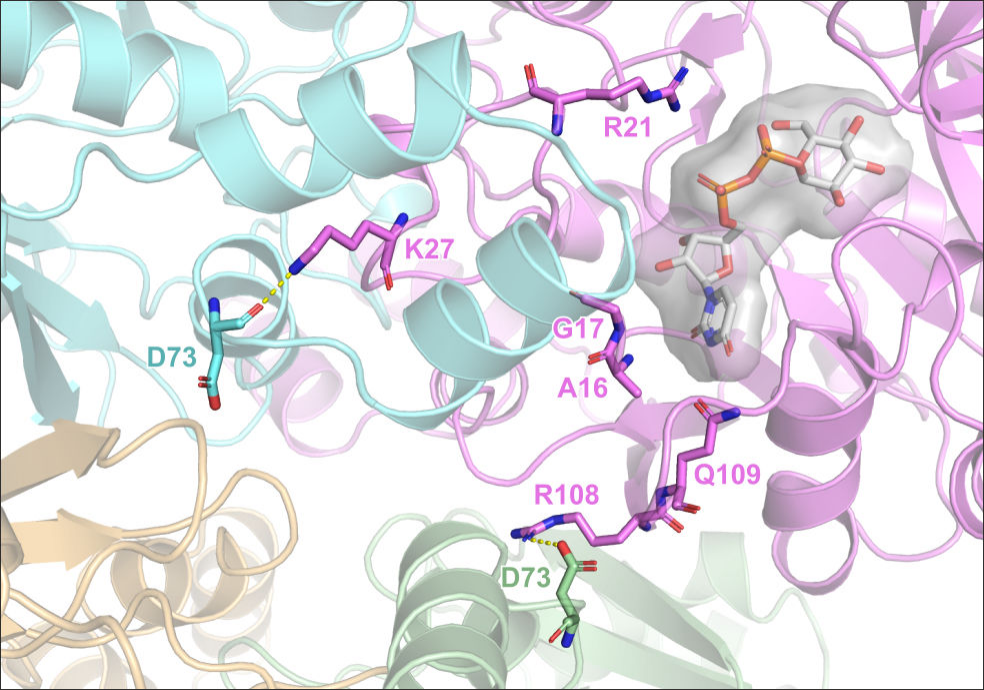
The proposed link between KpUGP tetramerization and catalysis. KpUGP tetramer AlphaFold model. For orientation, UDP-Glc from *P. aeruginosa* UGP (PDB 8F73), shown in semi-transparent stick and surface representation, was superimposed onto the active site of chain A (shown in magenta). Shown in stick conformation in the magenta subunit are active site residues A16, G17, R21, and Q109, as well as key residues for oligomerization, K27 and R108, which interact across the tight and loose dimer interfaces with the backbone and the sidechain of D73 from the cyan (chain B) and green (chain D) subunits, respectively.

Interestingly, the key residues that affected PaUGP tetramer stability and activity most severely (K21, D67, and R101) were strictly conserved in KpUGP (K27, D73, and R108) and appear to play the same roles. We propose that, like their homologs in PaUGP, these three residues mechanistically link KpUGP activity to tetramerization, as K27 and R108 are adjacent to crucial active site residues involved in substrate binding, and both appear to be stabilized in an intermolecular fashion by D73’s backbone (K27) and sidechain (R108) via the tight and loose dimer interface, respectively (Fig. 7). The discovery of this structurally and functionally conserved mechanism implies that it may be possible to develop compounds capable of inhibiting both KpUGP and PaUGP by interfering with their tetrameric assembly. Given the conservation of the involved residues across Gram-negative bacteria (Fig. S2), such compounds could have even broader applicability. A particularly interesting common target site could be the conserved residue KpUGP D73 (PaUGP D67), which is remote from the catalytic center (i.e., an allosteric site) but functionally crucial as it provides interactions affecting the active site across both dimeric interfaces (Fig. 7).

In conclusion, the results of our study underline that tetramerization is essential for enzymatic function of bacterial UGPs and is facilitated by a common mechanism utilizing conserved key residues. Targeting the respective molecular interfaces, which are absent in the human homolog, could provide a means of selectively inhibiting the bacterial virulence factor UGP and potentially rendering pathogenic species avirulent.

## MATERIALS AND METHODS

### AlphaFold models

Protein complex models of the KpUGP tetramer and KpGalF dimer were generated using the Multimer extension (66) of AlphaFold v2.3.1 software (67) with full genetic database configuration and without template structures. The query amino acid sequences were obtained from the corresponding Uniprot database entries: *K. pneumoniae* UGP—A0A377VZW9 and *K. pneumoniae* GalF—A0A377WNU3. All calculations were performed on the Halime HPC cluster at the Institute for Biophysical Chemistry at Hannover Medical School. For each complex, AlphaFold produced and ranked 25 energy-relaxed models. The models were superimposed using the secondary-structure matching tool (68) of WinCoot v0.987 EL (69). Due to the very high overall similarity between the models, only the best-ranked models of each protein (ranked_0) were chosen for detailed stereochemical analyses, which were carried out with PyMOL (The PyMOL Molecular Graphics System, Version 3.0 Schrödinger, LLC.) and UCSF ChimeraX (70, 71). The predicted local distance difference test, pTM, and ipTM scores, as well as Predicted Aligned Error plots (Fig. S12), were extracted from the AlphaFold output files. Illustrations were prepared with PyMOL (The PyMOL Molecular Graphics System, Version 3.0 Schrödinger, LLC.)

### Generation of KpUGP and KpGalF expression constructs

*K. pneumoniae galU* and *galF* were amplified from genomic DNA of the *K. pneumoniae* subsp. *pneumoniae* type strain DSM 30104 (also available under ATCC 13883 or NCTC 9633) obtained from DSMZ Braunschweig. This strain corresponds to *K. pneumoniae* capsular type K3 and is pathogenic to humans and animals; it does not produce extended spectrum β-lactamase and is sensitive to carbapenems. Both PCR products were digested via BamHI and XhoI restriction sites introduced via primers. The PCR product of *K. pneumoniae galU* was then ligated into a BamHI/XhoI digested, modified pET-22b expression vector (Novagen) containing an N-terminal Strep-tagII followed by a thrombin cleavage site (sequence: MASWSHPQFEKGALVPRGS), yielding the construct Strep-KpUGP. The *galF* PCR product was ligated into multiple cloning site (MCS) 1 of the BamHI/XhoI digested pETDuet-1 expression vector (Novagen) that contains an N-terminal His-tag, yielding the construct His-KpGalF. For direct comparison with KpGalF, an alternative expression construct for Strep-KpUGP in MCS1 of vector pETDuet-1 was created using the original Strep-KpUGP construct as a template. The PCR product and vector were digested with NcoI and EcoRI and ligated. The resulting purified Strep-KpUGP exhibited the same oligomeric state and activity as the protein resulting from the pET-22b vector.

### Point mutations and fusion PCR

Site-directed mutagenesis was performed according to Liu and Naismith (72) using primers listed in Table S3 and plasmids encoding wt Strep-KpUGP and His-KpGalF serving as PCR templates. Methylated template DNA was digested with DpnI after PCR. The integrity of all plasmids was confirmed by sequencing (Eurofins Genomics). For the KpGalF∆H1,H2+KpUGP H1,H2 construct, we individually amplified the sequence of wt His-KpGalF up until residue 267 as well as the last 32 residues of wt KpUGP, including the stop codon. In a third PCR, both PCR products were then fused and amplified using the cloning primers for KpGalF (fw) and KpUGP (rev; see Table S3).

### Recombinant expression of Strep-KpUGP and His-KpGalF

Ca^2+^-competent *E. coli* BL21(DE3) was transformed with the respective plasmid by heat shock and grown overnight at 37°C on lysogeny broth-agar plates supplemented with the appropriate antibiotic for selection. Protein expression was carried out according to the method developed by Ukkonen et al. (73) using the EnPresso B500 Kit (Enpresso) in a culture volume downscaled to 100 mL but otherwise according to the manufacturer’s instructions. Protein expression was induced with 1 mM isopropyl-1-thio-β-D-galactopyranoside. Bacteria were harvested by centrifugation (4,000 × *g*, 15 min, 4°C) and washed twice with PBS, and the pellets were stored at −20°C until further use.

### Protein purification by affinity chromatography

Expression culture pellets were resuspended in 25 mL of buffer W (100 mM Tris-HCl [pH 8.0] and 150 mM NaCl) for Strep-tagged proteins or buffer A (20 mM Na-phosphate[ pH 7.4] and 150 mM NaCl) for His-tagged proteins. Both buffers were supplemented with protease inhibitors (cOmplete ULTRA Tablets, Roche). Bacterial lysis was performed by sonication with a Branson sonifier (50% amplitude, 10 cycles of 30 s alternating with 30 s of rest) while being cooled on ice. The suspension was centrifuged (20,000 × *g*, 30 min, 4°C), and the supernatant passed through a 0.8 µL filter. The cleared lysate was loaded onto a 1 mL Strep-Tactin XT column (IBA) equilibrated with buffer W (for Strep-tagged proteins), or a 1 mL HisTrap HP column (Cytiva) equilibrated with buffer A with 20 mM imidazole (for His-tagged proteins). The columns were washed with 10 column volumes of the respective loading buffer and transferred to an ÄKTA system (Cytiva). Strep-tagged proteins were eluted with 100% buffer E (buffer W with 50 mM biotin). His-tagged proteins were eluted with a linear gradient of 20%–50% buffer B (buffer A with 500 mM imidazole) after removing unspecifically bound proteins with 7% buffer B. For all proteins, buffer exchange into 50 mM Tris-HCl [pH 8.0], 100 mM NaCl, and 10 mM MgCl_2_ was performed using a HiPrep 26/10 desalting column (Cytiva), and 1 mM dithiothreitol was added to the final protein preparation before flash freezing and storing aliquots at −80°C. Protein concentrations were determined from the absorbance at 280 nm and each protein’s specific extinction coefficient and molecular weight, calculated using ProtParam (http://web.expasy.org/protparam/).

### *In vitro* activity assay

UGP *in vitro* activity was determined using the EnzChek pyrophosphate assay (Invitrogen) at 25°C in 100 µL volume in 96-well half-area flat-bottom microplates (Greiner Bio-One). Substrate concentrations were 1 mM UTP and Glc-1-P each. Reactions were initiated by adding 10 µL of recombinant UGP/GalF in suitable dilution to 90 µL of reaction mastermix. Product formation was continuously measured for 4 min at 360 nm in a Power-Wave 340 microplate reader (Bio-Tek). To correct the data for background activity caused by (pyro)phosphate contaminations, control measurements initiated with 10 µL of buffer were performed. Statistical analyses were conducted using GraphPad Prism version 7.03 for Windows (GraphPad Software, Boston, MA, USA, https://www.graphpad.com/).

### SDS-PAGE analysis and immunoblotting

Protein integrity and purity were assessed by SDS-PAGE as described (33). His-tagged proteins were selectively visualized with horseradish peroxidase-anti-6× His tag antibody (abcam) and the Pierce ECL Western Blotting Substrate kit (Thermo scientific).

### Size exclusion chromatography

Protein apparent molecular weights were determined by size exclusion chromatography using a buffer composed of 50 mM Tris-HCl (pH 8.0), 300 mM NaCl, and 10 mM MgCl_2_ and a Superdex 200 10/300 GL column (GE healthcare) for KpUGP active site mutants. For KpUGP contact site mutants and the KpGalF constructs, a Superose 6 Increase 10/300 GL column (Cytiva) was used. Molecular weight marker proteins (Sigma) were subjected to chromatography under the same conditions for calibration.

### Blue native PAGE and in-gel activity staining

BN-PAGE was performed as previously described (33) with the following modifications: a total of 4.5 µg of each protein was loaded onto the gel, and after the alizarin red counter-staining, the gel was washed with 10% acetic acid and 30% ethanol to minimize background staining.

## SUPPLEMENTAL MATERIAL

Supplemental figures and tables mbio.02071-24-s0001.docx Figures S1-S11 and Tables S1-S3.

ASM does not own the copyrights to Supplemental Material that may be linked to, or accessed through, an article. The authors have granted ASM a non-exclusive, world-wide license to publish the Supplemental Material files. Please contact the corresponding author directly for reuse.
