# Supplementary material for: Identification and characterization of the functional tetrameric UDP-glucose pyrophosphorylase from Klebsiella pneumoniae
Source: mBio. 2024 Dec 20;16(2):e02071-24. doi: 10.1128/mbio.02071-24 (PMC11796359; doi:10.1128/mbio.02071-24)
Supplement: Supplemental figures and tables — Figures S1-S11 and Tables S1-S3. [file mbio.02071-24-s0001.docx]

# **Supplementary material**

# Identification and characterization of the functional tetrameric UDP‑glucose pyrophosphorylase from *Klebsiella pneumoniae*

**Isabel Ramón Roth^1^, Pavel Kats^2^, Timm Fiebig^1^, Françoise Routier^1^, Roman Fedorov^2,3^, Larissa Dirr^4^, Jana I. Führing^1🖂^**

^1^ Institute of Clinical Biochemistry, Hannover Medical School, Hannover, Germany

^2^ Institute for Biophysical Chemistry, Hannover Medical School, Hannover, Germany

^3^ Division for Structural Biochemistry, Hannover Medical School, Hannover, Germany

^4^ Institute for Biomedicine and Glycomics, Griffith University, Gold Coast, Southport, Australia

^🖂^ Corresponding author: Jana I. Führing [fuehring.jana@mh-hannover.de](mailto:fuehring.jana@mh-hannover.de)

# Table of contents

[Supplementary figure 1: 2](#_Toc179299686)

[Supplementary figure 2: 4](#_Toc179299687)

[Supplementary figure 3: 5](#_Toc179299688)

[Supplementary figure 4: 6](#_Toc179299689)

[Supplementary figure 5: 8](#_Toc179299690)

[Supplementary figure 6: 10](#_Toc179299691)

[Supplementary figure 7: 10](#_Toc179299692)

[Supplementary figure 8: 11](#_Toc179299693)

[Supplementary figure 9: 12](#_Toc179299694)

[Supplementary figure 10: 13](#_Toc179299695)

[Supplementary figure 11: 14](#_Toc179299696)

[Supplementary table 1: 16](#_Toc179299697)

[Supplementary table 2: 17](#_Toc179299698)

[Supplementary table 3: 18](#_Toc179299699)

# Supplementary figures


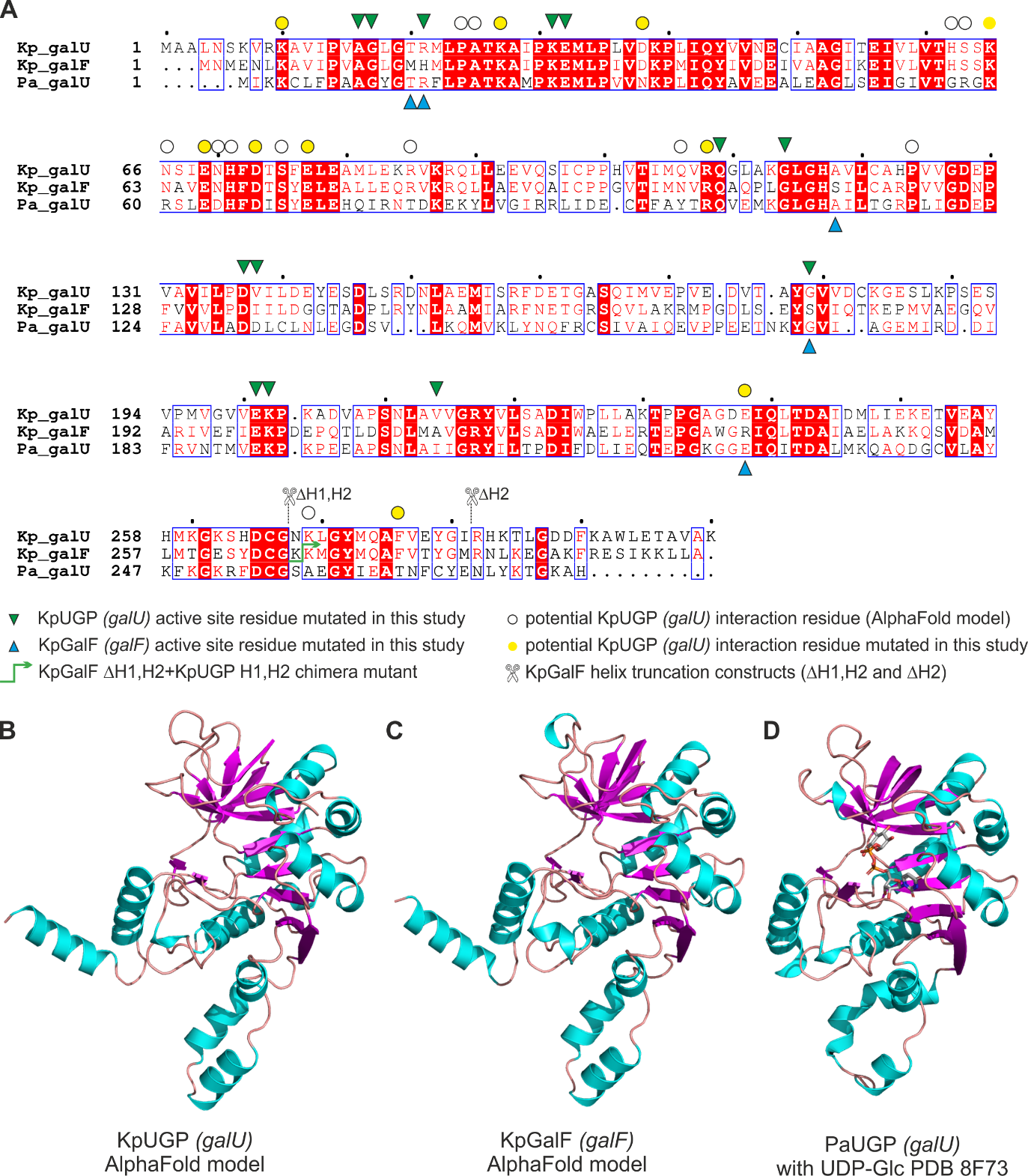


Supplementary figure 1: **Primary and tertiary structure comparison of *K. pneumoniae* UGP and GalF proteins with *P. aeruginosa* UGP.** (A) Sequence alignment of proteins encoded by Kp *galU* (KpUGP), Kp *galF* (KpGalF), and Pa *galU* (PaUGP). Active site residues are indicated by triangles and residues potentially mediating intermolecular contacts by circles. Colored triangles / circles indicate residues mutated in this study. Alignment created with MultAlin (Corpet, 1988). (B) – (D) Tertiary structures of KpUGP (AlphaFold model), KpGalF (AlphaFold model), PaUGP/UDP-Glc complex (PDB 8F73). A single subunit of each protein is shown in cartoon representation with α-helices, β-sheets, and loops colored in cyan, magenta, and pink, respectively. UDP-Glc in panel (D) is shown in stick presentation in grey, with oxygen, nitrogen, and phosphorus shown in red, blue, and orange, respectively.


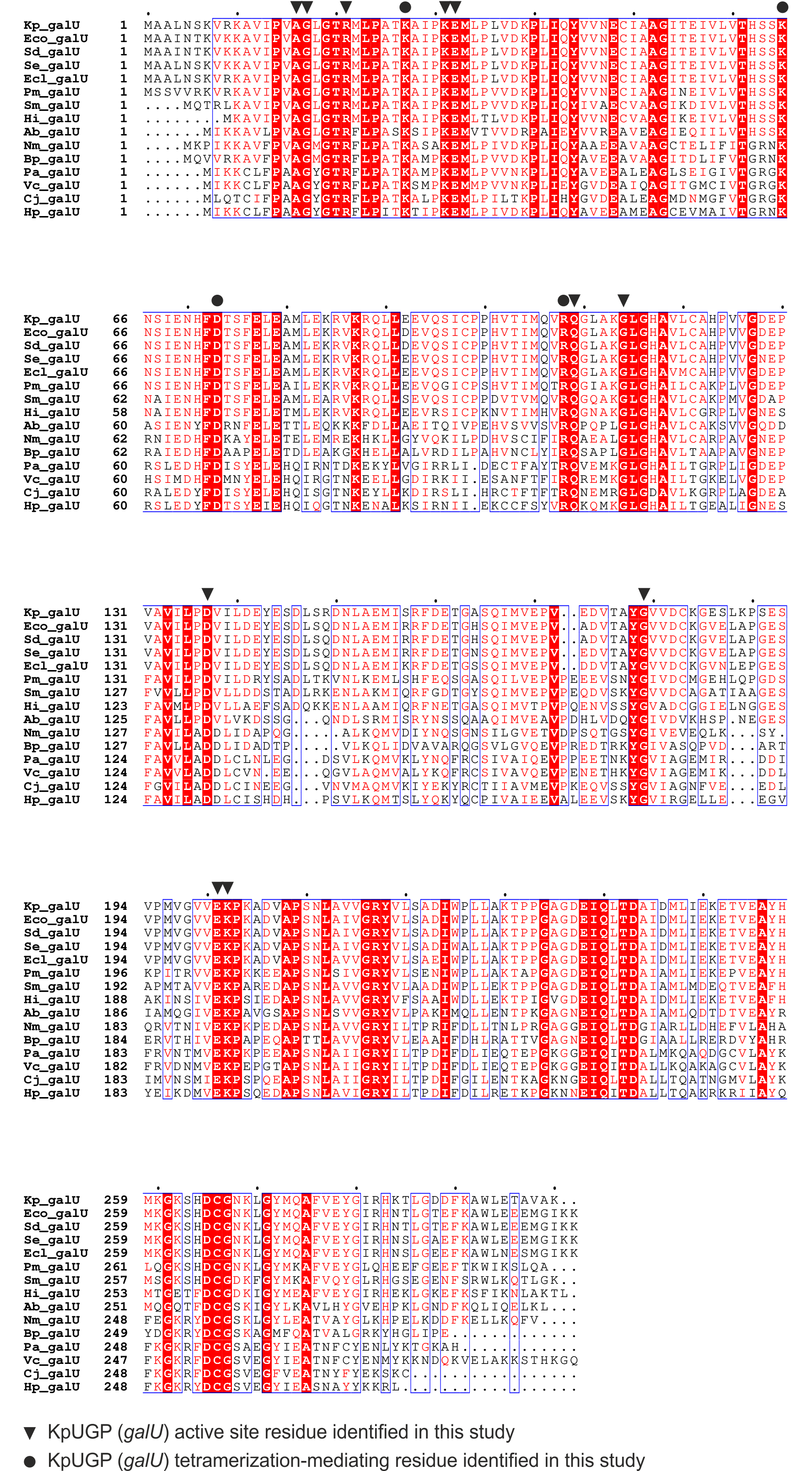


Supplementary figure 2: **Sequence alignment of UGP proteins (encoded by the *galU* gene) from clinically important Gram-negative bacteria.** Kp, *Klebsiella pneumoniae*; Eco, *Escherichia coli*; Sd, *Shigella dysenteriae*; Se, *Salmonella enterica*; Ecl, *Enterobacter cloacae*; Pm, *Proteus mirabilis*; Sm, *Serratia marcescens*; Hi, *Haemophilus influenzae*; Ab, *Acinetobacter baumannii*; Nm, *Neisseria* *meningitidis*; Bp, *Bordetella* *pertussis*; Pa, *Pseudomonas* *aeruginosa*; Vc, *Vibrio* *cholera*; Cj, *Campylobacter* *jejuni*; Hp, *Helicobacter* *pylori*. Alignment created with MultAlin (Corpet, 1988).


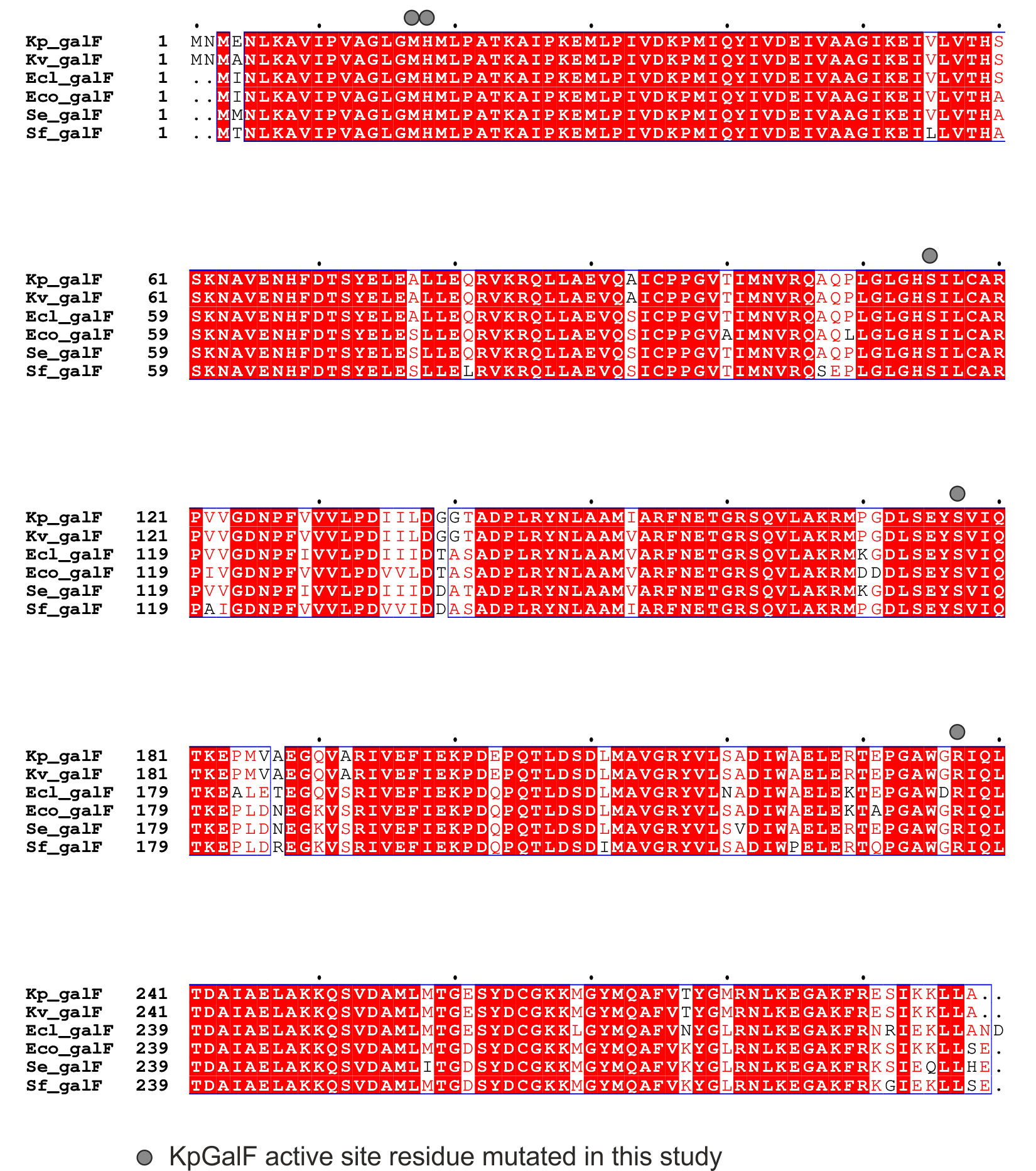


Supplementary figure 3: **Sequence alignment of enterobacterial GalF proteins.** Kp, *Klebsiella* *pneumoniae*; Kv, *Klebsiella* *variicola*; Ecl, *Escherichia* *coli*; Eco, *Enterobacter* *cloacae*; Se, *Salmonella* *enterica*; Sf, *Shigella* *flexneri*. Alignment created with MultAlin (Corpet, 1988).


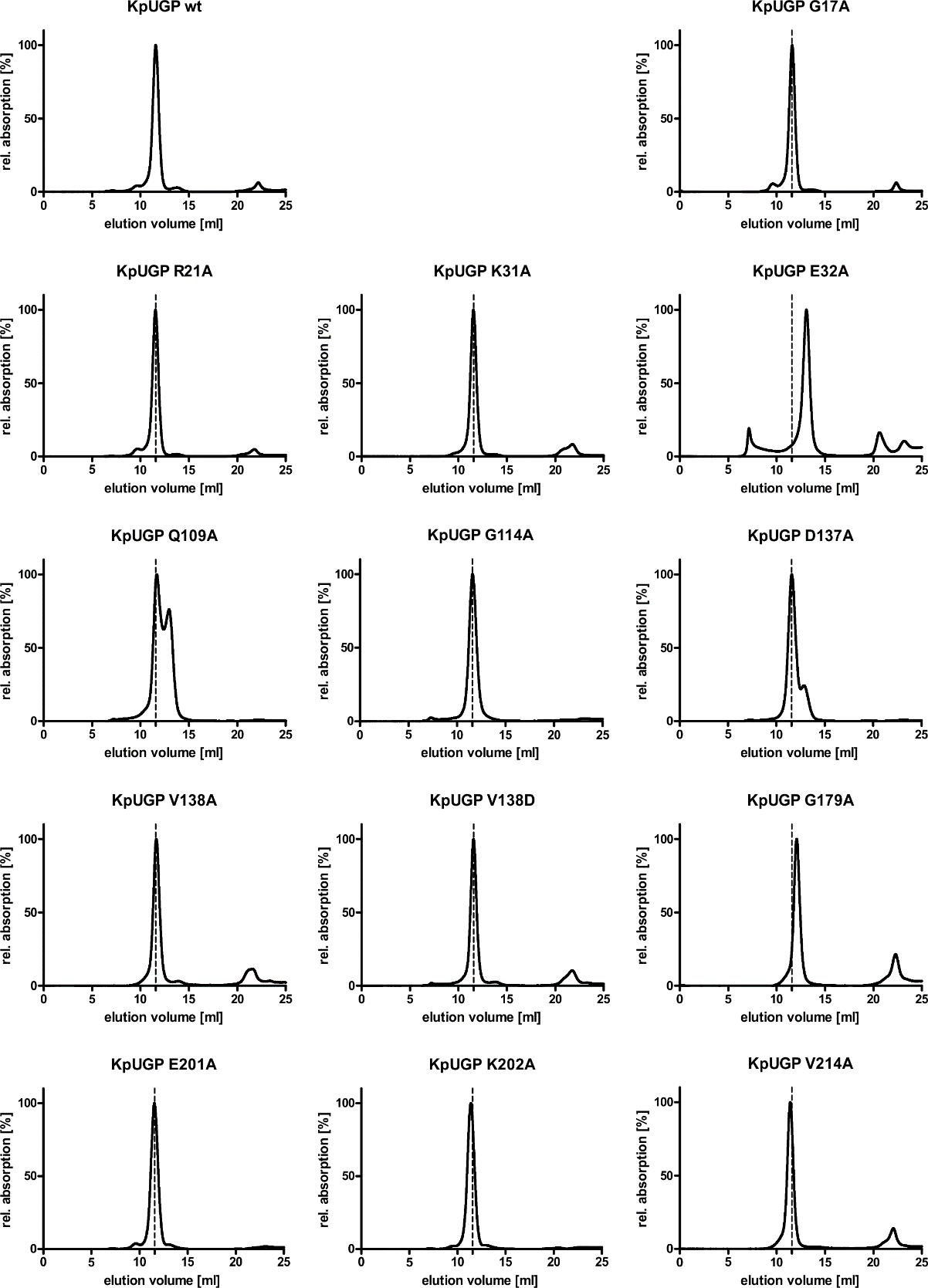


Supplementary figure 4: **Size exclusion chromatography (SEC) elution profiles of KpUGP active site mutants.** SEC was performed using a Superdex 200 10/300 GL column (GE healthcare). The dashed vertical line corresponds to the elution volume of tetrameric wild-type KpUGP for reference.


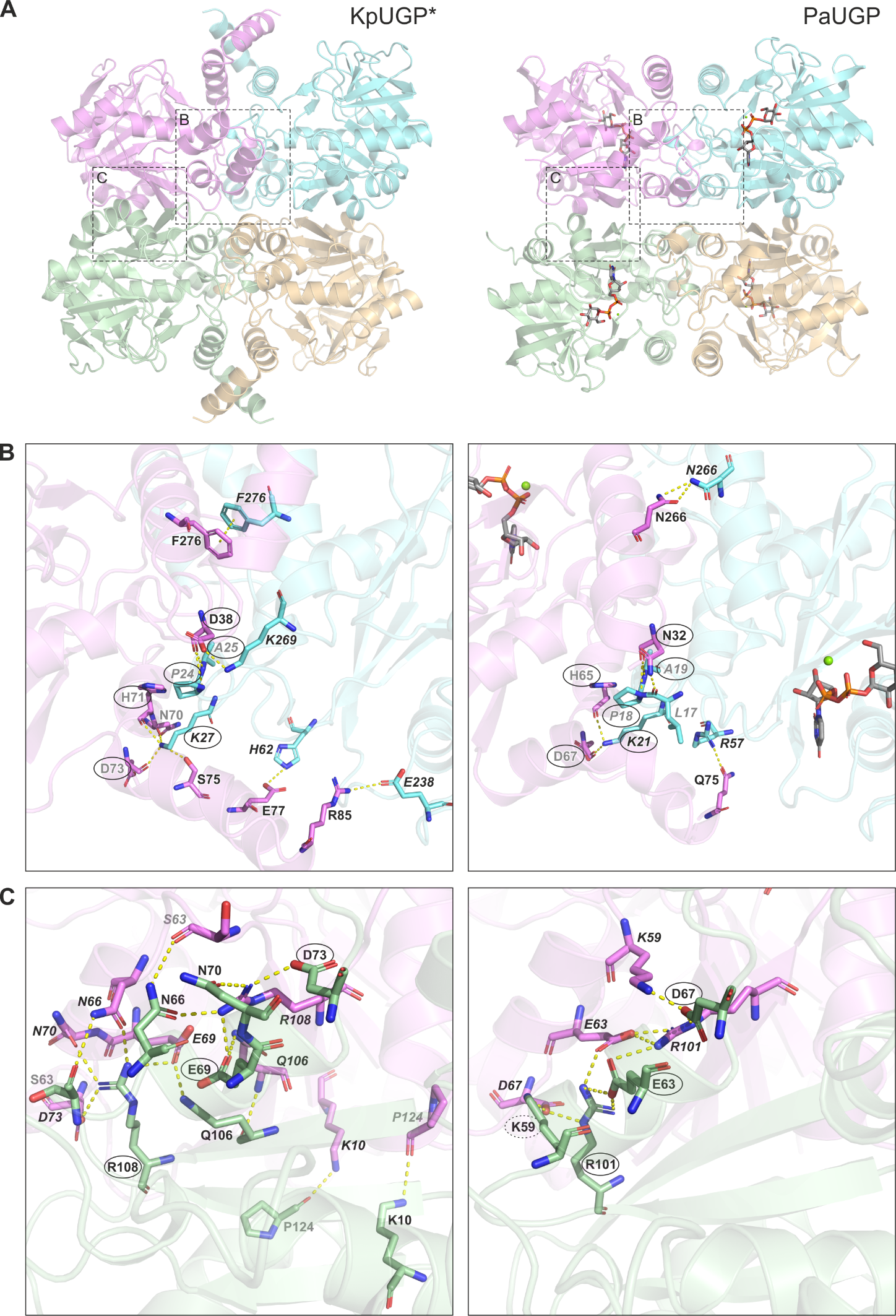


Supplementary figure 5: **Comparison of tetrameric assembly in KpUGP (left) and PaUGP (right).** (A) Tetrameric KpUGP (AlphaFold model) and PaUGP in complex with UDP-Glc (PDB 8F73) with subunits A, B, C and D shown in pink, cyan, gold, and green, respectively. (B) Interactions at the tight dimer interface between subunits A and B, and (C) mutual interactions at the loose dimer interface between subunits A and D. Potential interacting residues are shown in stick representation and labeled; residues interacting via their backbones are labeled in grey. Oxygen, nitrogen, phosphorus, and magnesium are shown in red, blue, orange, and green, respectively. Interactions are shown as yellow dotted lines. Residues conserved or functionally similar between KpUGP and PaUGP are circled.

**
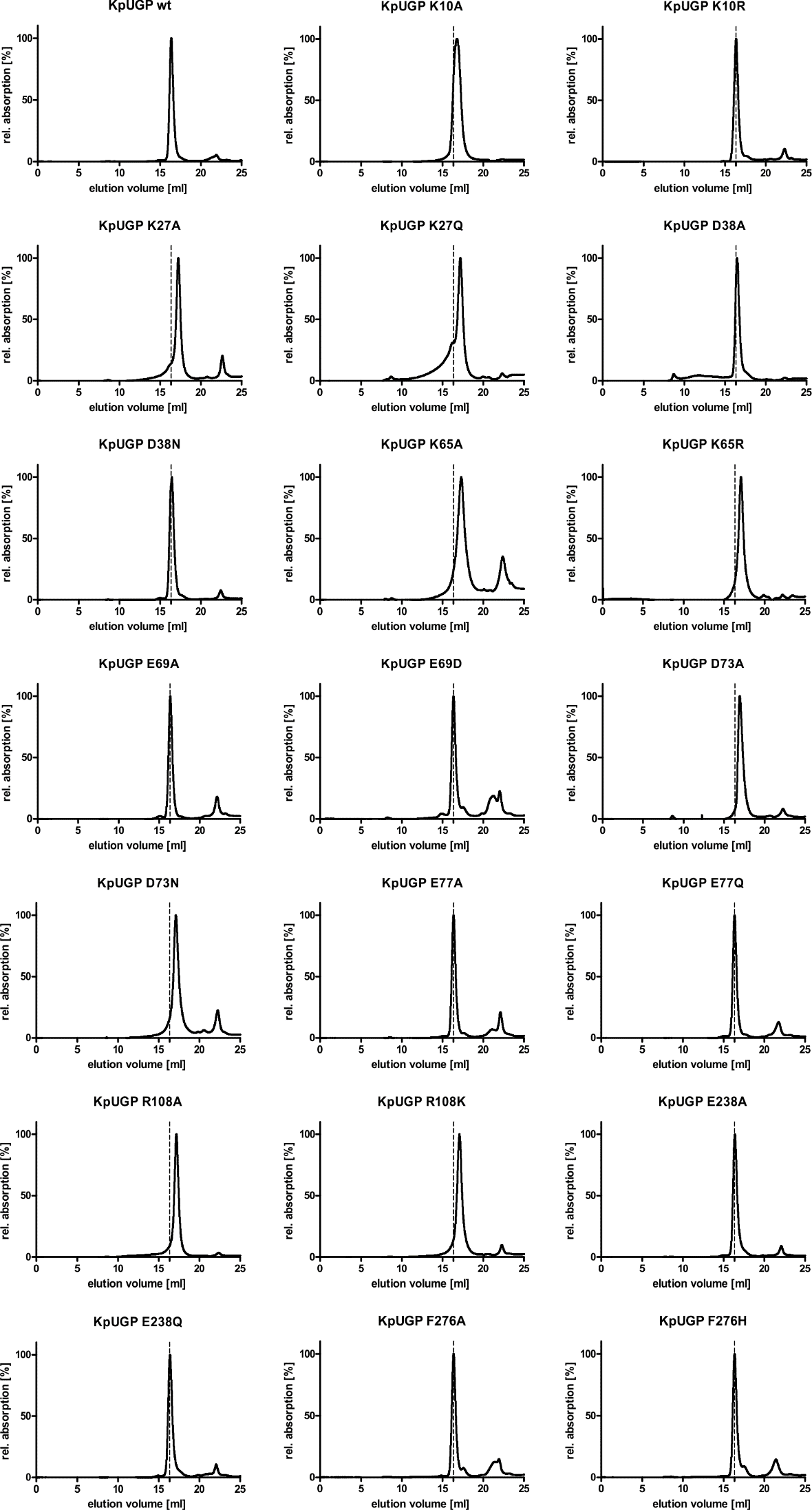
**

Supplementary figure 6: **Size exclusion chromatography (SEC) elution profiles of KpUGP oligomerization interface mutants.** SEC was performed using a Superose 6 Increase 10/300 GL column (Cytiva). The dashed vertical line corresponds to the elution volume of tetrameric wild-type KpUGP for reference.


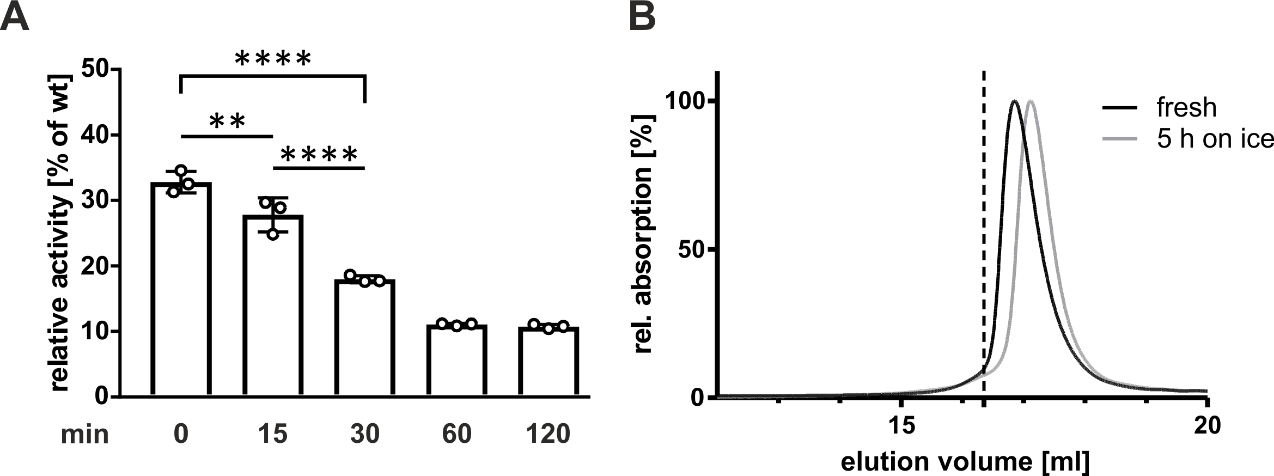


Supplementary figure 7: **Time-dependent inactivation and dissociation of KpUGP D73A.** (A) Time-dependent loss of enzymatic activity (expressed as % of wt activity, which was defined as 100%) of KpUGP D73A. Individual values, means and standard deviation for three technical replicates are plotted. One-way ANOVA indicated significant differences (p < 0.0001) and selected results from Tukey’s post hoc test are shown (**p < 0.01, ****p < 0.0001), indicating progressive loss of enzymatic activity over time. min = minutes of incubation time on ice after thawing. (B) Size exclusion chromatography elution profiles of KpUGP D73A directly after thawing (“fresh”) and after 5 h incubation on ice. For uniform representation, the maximum peak intensity was set to 100%, resulting in the relative absorption shown in the y-axis. The dashed vertical line corresponds to the elution volume of tetrameric wild-type KpUGP for reference.


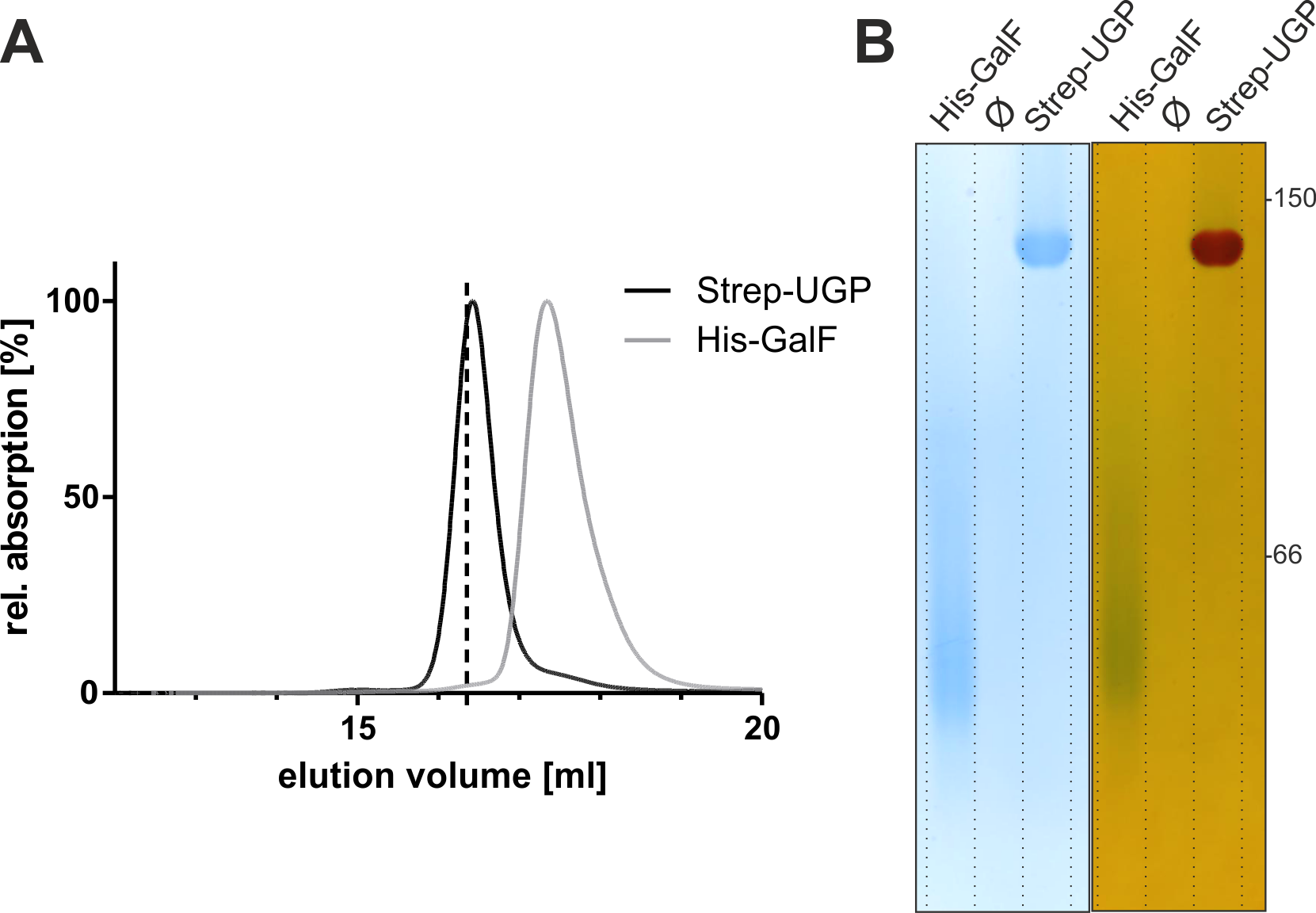


Supplementary figure 8: **Comparative analysis of *K. pneumoniae* UGP (Strep-tagged) and GalF (His-tagged).** Both proteins were expressed in the pETDuet-1 vector. (A) SEC elution profiles of His-GalF and Strep-UGP. The calculated oligomerization states are given in Supplementary table 1. For comparison, the elution volume of Strep-UGP derived from the pET-22b expression construct is shown as a dashed vertical line. (B) Blue Native PAGE and in-gel activity staining of His-GalF and Strep‑UGP. ∅ indicates empty lanes.


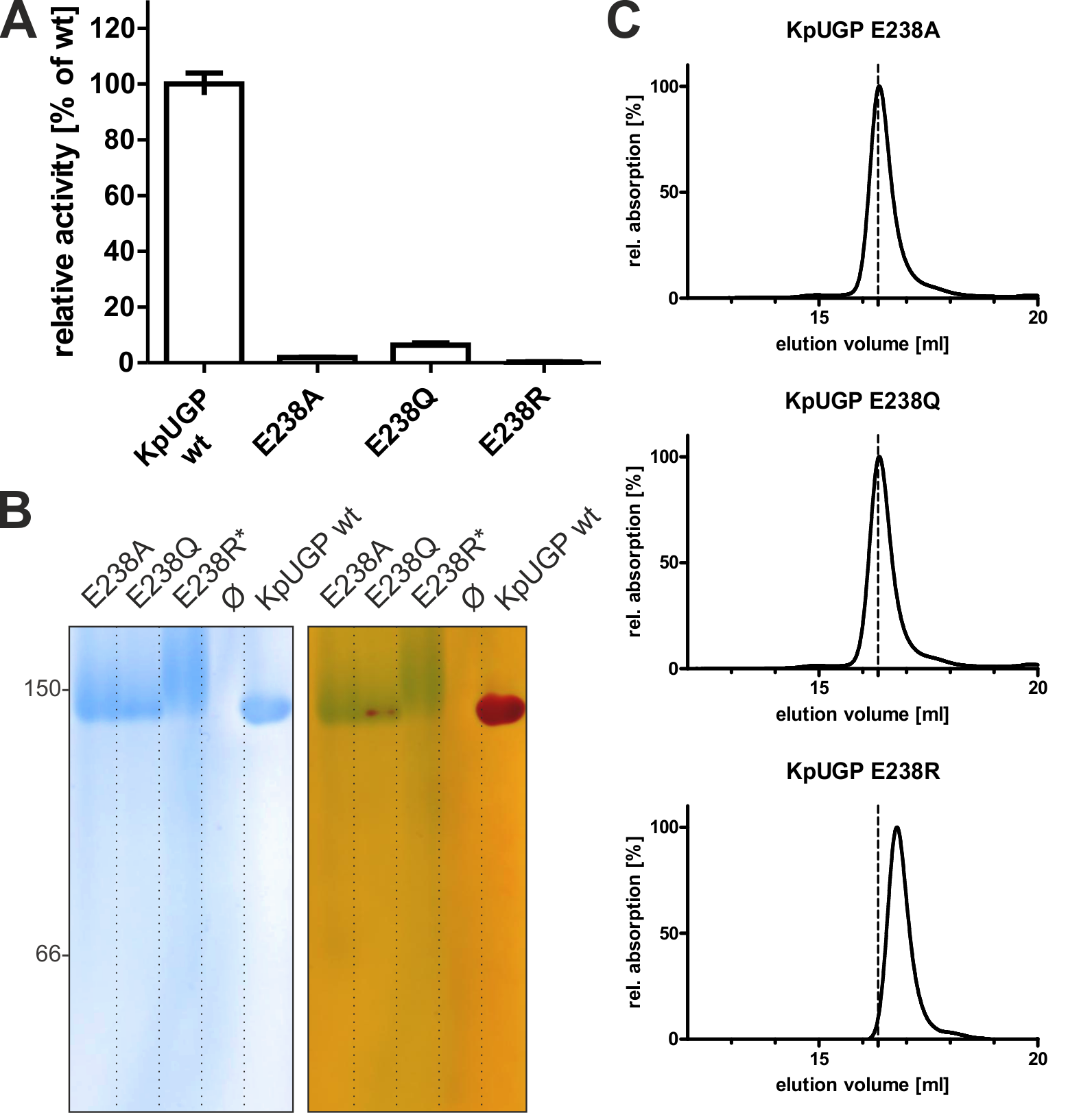


Supplementary figure 9: **Enzymatic activity and oligomeric states of KpUGP E238 mutants.** (A) *In vitro* activity in % of KpUGP wt. Activities were determined in the forward reaction (UDP-Glc synthesis) and are shown as means ± SEM of at least three independent experiments, each performed in technical triplicate, and expressed as % of wt activity, which was defined as 100%. (B) Blue Native PAGE and subsequent activity staining; ∅ indicates empty lanes. (C) SEC profiles of KpUGP E238 mutants; the dashed vertical line corresponds to the elution volume of tetrameric wild-type KpUGP for reference.


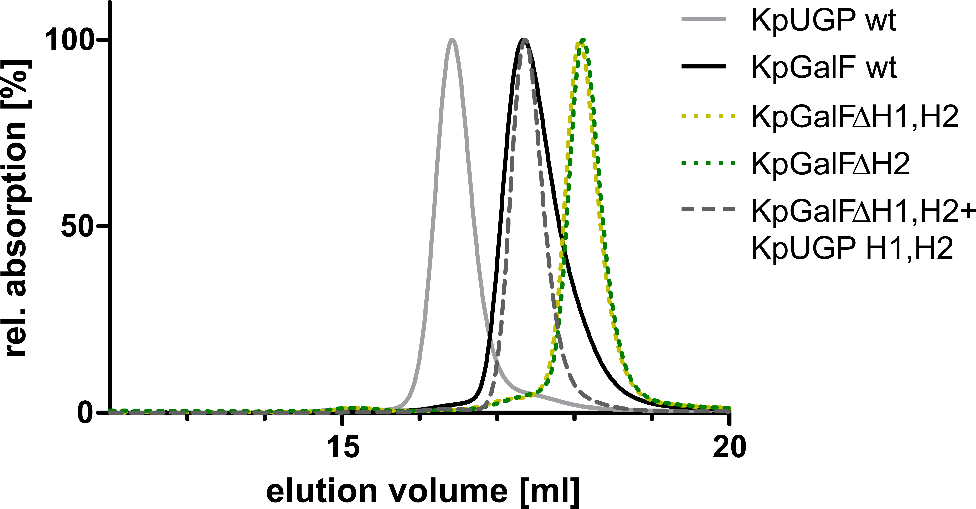


Supplementary figure 10: **Influence of C-terminal α-helices on KpGalF oligomerization.** Size exclusion chromatography elution profiles of tetrameric wild-type KpUGP and dimeric wild-type KpGalF are shown for comparison. KpGalF∆H1,H2: KpGalF lacking last two α-helices; KpGalF∆H2: KpGalF lacking the terminal α-helix; KpGalF∆H1,H2+KpUGP H1,H2: KpGalF whose last two α‑helices have been replaced by those of KpUGP. See Supplementary figure 1A for details on the primary sequence of the truncation and hybrid constructs.


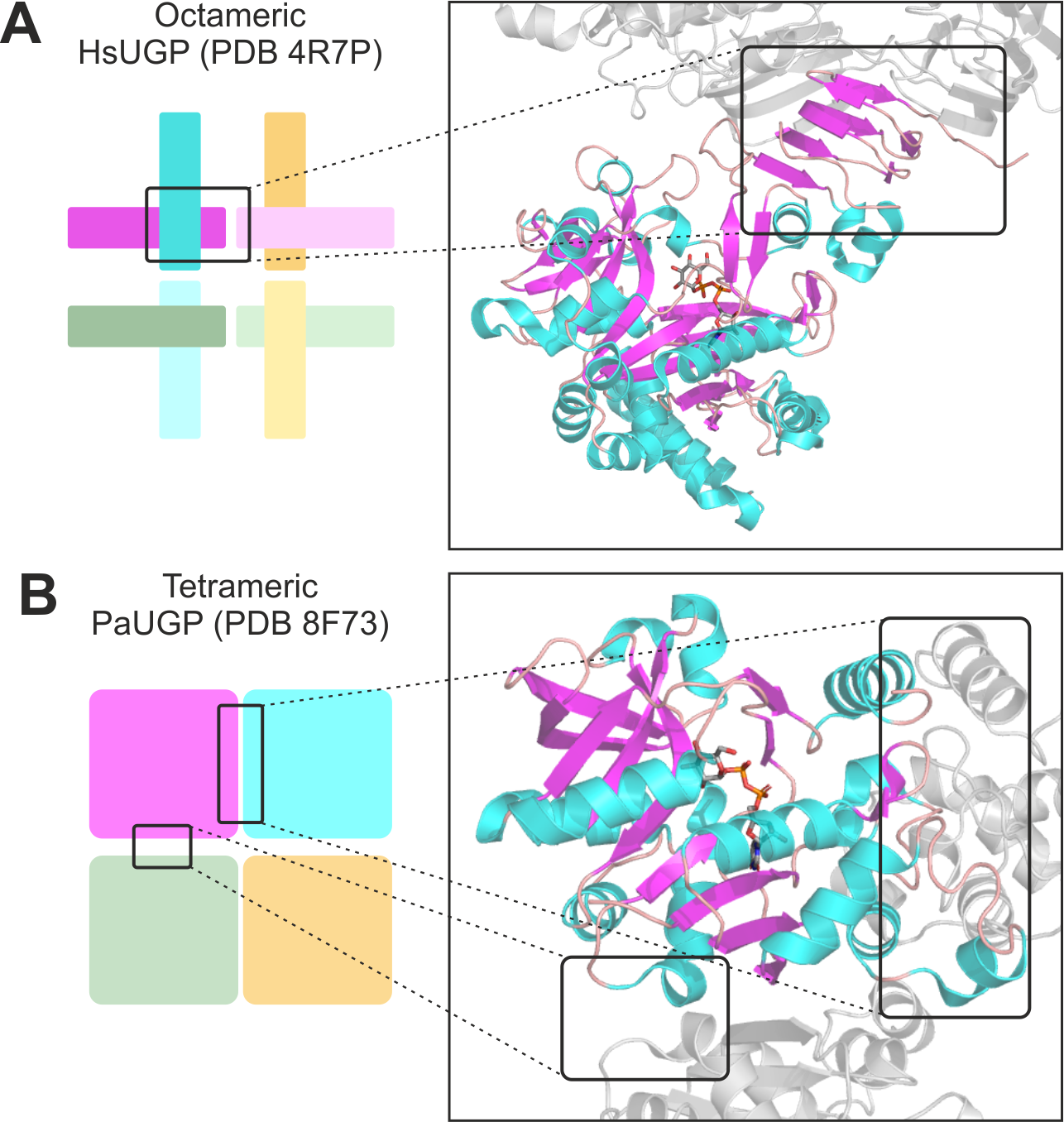


Supplementary figure 11: **Comparison of (A) octameric *Homo sapiens* UGP (HsUGP; PDB 4R7P) and (B) tetrameric bacterial UGP (PaUGP; PDP 8F73).** The figure illustrates how human and bacterial UGPs use vastly different modes of oligomerization, which are facilitated by unique structural elements. The left side of each panel shows schematic representations of the octameric assembly of HsUGP and the tetrameric assembly of PaUGP, respectively, with framed oligomerization interfaces. In HsUGP (a tetramer of dimers), the magenta and cyan subunits form side-by-side dimers, and the magenta and light pink subunits form end-to-end dimers. In PaUGP (a dimer of dimers), the magenta and cyan subunits form a tight dimer, and the magenta and green subunits form a loose dimer. The right side of each panel shows a cartoon representation of the respective magenta subunit, with bound product, in the same orientation. UDP-Glc is shown in stick representation; α-helices, β-sheets and loops are shown in cyan, magenta, and pink, respectively. Shown transparent in grey are the respective two partner subunits forming the end-to-end and side-by-side dimer in HsUGP, and the tight and loose dimer partner subunits in PaUGP. All other subunits of the octa- and tetramer, respectively, are omitted for clarity.

**Supplementary figure 12: Predicted Aligned Error (PAE) plots for AlphaFold models** of (A) the KpUGP tetramer, (B) the KpGalF dimer.


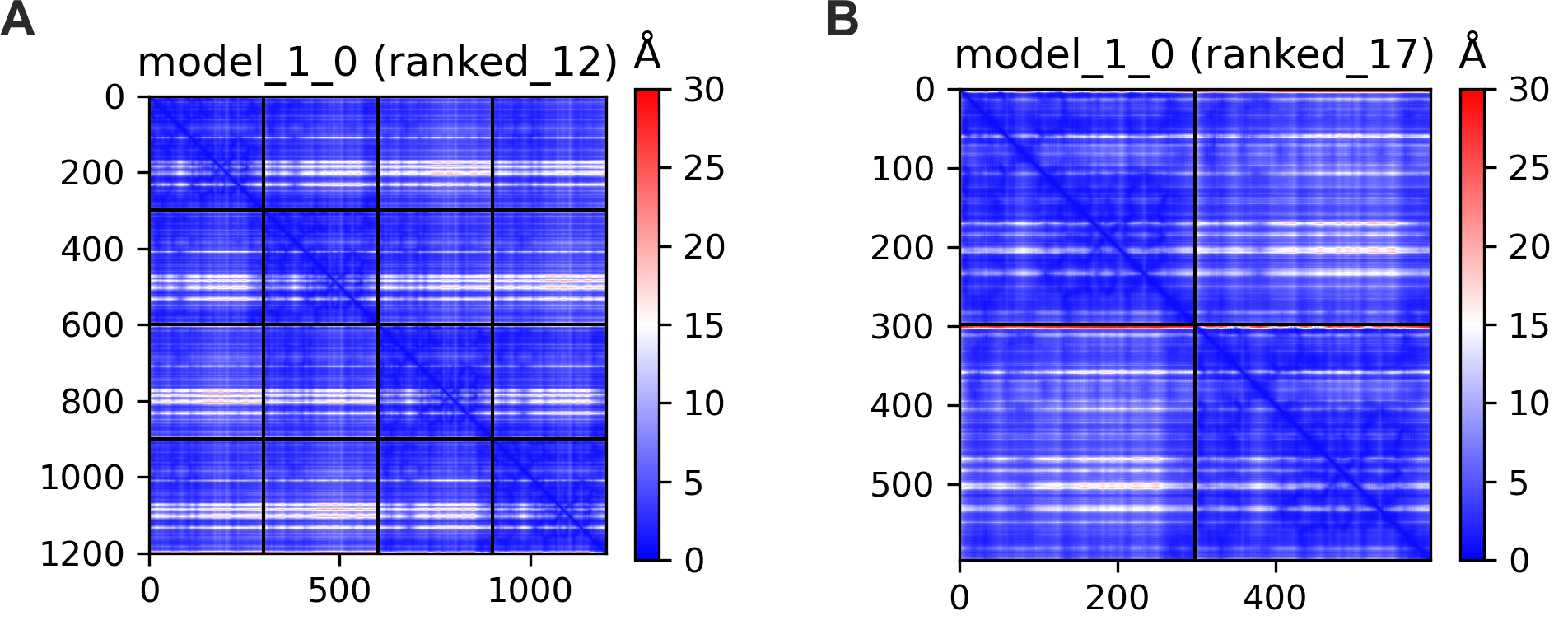


# Supplementary tables

Supplementary table 1: **Enzymatic activity and oligomeric state of wild-type KpUGP, wild-type KpGalF and mutant KpGalF.** All proteins were expressed in the pETDuet-1 vector. Activities were determined in the forward reaction (UDP-Glc synthesis) and are given as means ± SEM of at least three independent experiments, each performed in technical triplicate, and expressed as % of wt activity which was defined as 100%. Size exclusion chromatography (SEC) was performed using a Superose 6 Increase 10/300 GL column (Cytiva). Oligomeric states were calculated based on elution volumes of proteins of known size.

| **Protein** | **Activity**  **[% of KpUGP wt]** | **Elution volume [mL]** | **Oligomeric state** |
| --- | --- | --- | --- |
| wild-type KpUGP | 100 ± 10.46 | 16.42 | 4.3 |
| wild-type KpGalF | 0.06 ± 0.01 | 17.34 | 1.9 |
| KpGalF quintuple revertant mutant M17T/H18R/S115A/S177G/R237E | 1.62 ± 0.13 | 17.51 | 1.7 |

Supplementary table 2: **Elution volumes and oligomeric states of KpUGP active site mutants.** Size exclusion chromatography (SEC) was performed using a Superdex 200 10/300 GL column (GE healthcare). Oligomeric states were calculated based on elution volumes of proteins of known size. Where multiple protein peaks were observed, the main peak is given in bold print.

| **Protein** | **Elution volume(s) [mL]** | **Oligomeric state(s)** |
| --- | --- | --- |
| wt | 11.58 | 4.9 |
| G17A | 11.61 | 4.8 |
| R21A | 11.56 | 4.9 |
| K31A | 11.55 | 4.9 |
| E32A | 13.08 | 2.5 |
| Q109A | **11.69**  12.95 | **4.6**  2.6 |
| G114A | 11.60 | 4.8 |
| D137A | **11.60**  12.86 | **4.8**  2.7 |
| V138A | 11.64 | 4.7 |
| V138D | 11.56 | 4.9 |
| G179A | 12.07 | 3.9 |
| E201A | 11.55 | 4.9 |
| K202A | 11.39 | 5.3 |
| V214A | 11.41 | 5.3 |

Supplementary table 3: **Mutagenesis and cloning primers and their sequences.** Altered bases for site-directed mutagenesis are printed in lowercase; fw, forward; rev, reverse. Underlined sequences denote the overhang used for fusing PCR products.

| Description | Primer sequence (5’-3’) |
| --- | --- |
| Cloning primer Strep-KpUGP in pET-22b fw | CGTGGATCCGCTGCCCTTAATTCTAAAGTCAG |
| Cloning primer Strep-KpUGP in pET-22b rev | GTGCTCGAGTTACTTCGCTACCGCC |
| KpUGP K10A fw | GTCAGAgcAGCGGTTATCCCGGTTGCGG |
| KpUGP K10A rev | TAACCGCTgcTCTGACTTTAGAATTAAGGGCAGCCA |
| KpUGP K10R fw | GTCAGAAgAGCGGTTATCCCGGTTGCGG |
| KpUGP K10R rev | TAACCGCTcTTCTGACTTTAGAATTAAGGGCAGCCA |
| KpUGP G17A fw | CCGGTTGCGGcATTAGGCACCAGGATGCTGCCGG |
| KpUGP G17A rev | GGTGCCTAATgCCGCAACCGGGATAACCGCTTTTCTGACTTTAG |
| KpUGP R21A fw | TTAGGCACCgcGATGCTGCCGGCCACCAAAGCGATTC |
| KpUGP R21A rev | CGGCAGCATCgcGGTGCCTAATCCCGCAACCGG |
| KpUGP K27A fw | CCACCgcAGCGATTCCAAAAGAAATGCTGCCGCTGGTAG |
| KpUGP K27A rev | GAATCGCTgcGGTGGCCGGCAGCATCCTGGTG |
| KpUGP K27Q fw | CCGGCCACCcAAGCGATTCCAAAAGAAATGCTGCCGC |
| KpUGP K27Q rev | TGGAATCGCTTgGGTGGCCGGCAGCATCCTGGTGC |
| KpUGP K31A fw | GCGATTCCAgcAGAAATGCTGCCGCTGGTAGATAAGCC |
| KpUGP K31A rev | CAGCATTTCTgcTGGAATCGCTTTGGTGGCCGGC |
| KpUGP E32A fw | ATTCCAAAAGcAATGCTGCCGCTGGTAGATAAGCCGTTAATTC |
| KpUGP E32A rev | CGGCAGCATTgCTTTTGGAATCGCTTTGGTGGCC |
| KpUGP D38A fw | CTGGTAGcTAAGCCGTTAATTCAGTACGTTGTTAATGAATG |
| KpUGP D38A rev | CGGCTTAgCTACCAGCGGCAGCATTTCTTTTGG |
| KpUGP D38N fw | CTGGTAaATAAGCCGTTAATTCAGTACGTTGTTAATGAATG |
| KpUGP D38N rev | CGGCTTATtTACCAGCGGCAGCATTTCTTTTGG |
| KpUGP K65A fw | CTCGTCGgcAAACTCTATCGAAAACCATTTCGATAC |
| KpUGP K65A rev | GTTTgcCGACGAGTGCGTTACCAGAACAATTTC |
| KpUGP K65R fw | CTCGTCGAgAAACTCTATCGAAAACCATTTCGATAC |
| KpUGP K65R rev | GTTTctCGACGAGTGCGTTACCAGAACAATTTC |
| KpUGP E69A fw | AACTCTATCGcAAACCATTTCGATACCAGTTTTGAGCTGG |
| KpUGP E69A rev | GAAATGGTTTgCGATAGAGTTTTTCGACGAGTGCGTTAC |
| KpUGP E69D fw | AACTCTATCGAcAACCATTTCGATACCAGTTTTGAGCTGG |
| KpUGP E69D rev | GAAATGGTTgTCGATAGAGTTTTTCGACGAGTGCGTTAC |
| KpUGP D73A fw | AACCATTTCGcTACCAGTTTTGAGCTGGAAGCGATG |
| KpUGP D73A rev | AAAACTGGTAgCGAAATGGTTTTCGATAGAGTTTTTCGACG |
| KpUGP D73N fw | AACCATTTCaATACCAGTTTTGAGCTGGAAGCGATG |
| KpUGP D73N rev | AAAACTGGTATtGAAATGGTTTTCGATAGAGTTTTTCGACG |
| KpUGP E77A fw | ACCAGTTTTGcGCTGGAAGCGATGCTGGAAAAACGTGTAAAAC |
| KpUGP E77A rev | CGCTTCCAGCgCAAAACTGGTATCGAAATGGTTTTCGATAGAG |
| KpUGP E77Q fw | ACCAGTTTTcAGCTGGAAGCGATGCTGGAAAAACGTGTAAAAC |
| KpUGP E77Q rev | CGCTTCCAGCTgAAAACTGGTATCGAAATGGTTTTCGATAGAG |
| KpUGP R108A fw | CAAGTTgcTCAGGGCCTGGCGAAAGGTCTGGGTCACG |
| KpUGP R108A rev | CGCCAGGCCCTGAgcAACTTGCATAATAGTCACGTGCGG |
| KpUGP K108K fw | CAAGTTaagCAGGGCCTGGCGAAAGGTCTGGGTCACG |
| KpUGP R108K rev | CGCCAGGCCCTGcttAACTTGCATAATAGTCACGTGCGG |
| KpUGP Q109A fw | CAAGTTCGTgcGGGCCTGGCGAAAGGTCTGGGTCACG |
| KpUGP Q109A rev | CGCCAGGCCCgcACGAACTTGCATAATAGTCACGTGCGG |
| KpUGP G114A fw | CTGGCGAAAGcTCTGGGTCACGCGGTGCTGTGC |
| KpUGP G114A rev | GTGACCCAGAgCTTTCGCCAGGCCCTGACGAACTTG |
| KpUGP D137A fw | ATTCTGCCAGcCGTTATTCTGGATGAATATGAATCCGATCTGAG |
| KpUGP D137A rev | CAGAATAACGgCTGGCAGAATAACCGCGACCGG |
| KpUGP V138A fw | CCAGACGcTATTCTGGATGAATATGAATCCGATCTGAG |
| KpUGP V138A rev | CAGAATAGCGTCTGGCAGAATAACCGCGACC |
| KpUGP V138D fw | CCAGACGaTATTCTGGATGAATATGAATCCGATCTGAG |
| KpUGP V138D rev | CAGAATATCGTCTGGCAGAATAACCGCGACC |
| KpUGP G179A fw | ATGcCGTGGTTGACTGCAAAGGCGAATC |
| KpUGP G179A rev | CAACCACGgCATAAGCGGTTACGTCTTCG |
| KpUGP E201A fw | GGCGTGGTTGcGAAGCCAAAAGCTGACGTTGCGC |
| KpUGP E201A rev | TTTTGGCTTCgCAACCACGCCCACCATTGGTACGCTTTC |
| KpUGP K202A fw | GTGGTTGAGgcGCCAAAAGCTGACGTTGCGCCG |
| KpUGP K202A rev | AGCTTTTGGCgcCTCAACCACGCCCACCATTGGTAC |
| KpUGP V214A fw | GGCGGcGGTGGGGCGTTATGTCCTCAG |
| KpUGP V214A rev | CACCgCCGCCAGGTTGGACGGCG |
| KpUGP E238A fw | GGTGATGcAATTCAGCTGACTGACGCCATTGATATG |
| KpUGP E238A rev | GCTGAATTgCATCACCAGCGCCCGGAG |
| KpUGP E238Q fw | GGTGATcAAATTCAGCTGACTGACGCCATTGATATG |
| KpUGP E238Q rev | GCTGAATTTgATCACCAGCGCCCGGAG |
| KpUGP E238R fw | GGTGATagAATTCAGCTGACTGACGCCATTGATATG |
| KpUGP E238R rev | GCTGAATTctATCACCAGCGCCCGGAG |
| KpUGP F276A fw | GGCGgcTGTCGAATATGGTATTCGTCACAAAACGC |
| KpUGP F276A rev | ATTCGACAgcCGCCTGCATGTAGCCAAGCTTATTA |
| KpUGP F276H fw | GGCGcaTGTCGAATATGGTATTCGTCACAAAACGC |
| KpUGP F276H rev | ATTCGACAtgCGCCTGCATGTAGCCAAGCTTATTA |
| Cloning primer His-KpGalF in pETDuet-1 (MCS1) fw | GCTAGGATCCCAATATGGAGAATTTGAAAGCGG |
| Cloning primer His-KpGalF in pETDuet-1 (MCS1) rev | GCTAGCGGCCGCTCAGGCCAGCAGTTT |
| KpGalF M17T, H18R, S115A, S177G, R237E fw | ACTAGGCAcGCgTATGCTGCCGGCC |
| KpGalF M17T, H18R, S115A, S177G, R237E rev | ATAcGCgTGCCTAGTCCTGCGACCGGAATAA |
| KpGalF M17T, H18R, S115A, S177G, R237E fw | GCCACgCCATCCTCTGCGCCCGG |
| KpGalF M17T, H18R, S115A, S177G, R237E rev | GGATGGcGTGGCCCAGGCCCAGCG |
| KpGalF M17T, H18R, S115A, S177G, R237E fw | CGAATACggCGTCATCCAGACCAAAGAGCCGATG |
| KpGalF M17T, H18R, S115A, S177G, R237E rev | GATGACGccGTATTCGGAAAGATCGCCCGGC |
| KpGalF M17T, H18R, S115A, S177G, R237E fw | GGGCgagATCCAACTGACCGACGCC |
| KpGalF M17T, H18R, S115A, S177G, R237E rev | GGATctcGCCCCATGCGCCCGG |
| KpGalF K267Stop (KpGalF∆H1,H2) fw | GCGGGtAGAAAATGGGCTACATGCAGGC |
| KpGalF K267Stop (KpGalF∆H1,H2) rev | CATTTTCTaCCCGCAGTCGTAGCTTTCGCC |
| KpGalF K281Stop (KpGalF∆H2) fw | GGATGtGaAACCTGAAAGAAGGCGCCAAATTC |
| KpGalF K281Stop (KpGalF∆H2) rev | AGGTTtCaCATCCCGTAGGTGACGAAGGC |
| KpGalF∆H1,H2+KpUGP H1,H2 fw primer for KpGalF segment | CTACGACTGCGGGAAGAAGCTTGGCTACATGCAG |
| KpGalF∆H1,H2+KpUGP H1,H2 rev primer for KpGalF segment | GCTAGCGGCCGCTTACTTCGCTACCGCC |
| KpGalF∆H1,H2+KpUGP H1,H2 fw primer for KpUGP segment | GCTAGGATCCCAATATGGAGAATTTGAAAGCGG |
| KpGalF∆H1,H2+KpUGP H1,H2 rev primer for KpUGP segment | GTAGCCAAGCTTCTTCCCGCAGTCGTAG |
| Cloning primer Strep-KpUGP in pETDuet-1 (MCS1) fw | GCTACCATGGCTAGCTGGAGC |
| Cloning primer Strep-KpUGP in pETDuet-1 (MCS1) rev | GCTAGAATTCTTACTTCGCTACCGCC |
